# Application of ATR-FTIR and FT-NIR spectroscopy coupled with chemometrics for species identification and quality prediction of boletes

**DOI:** 10.1016/j.fochx.2024.101661

**Published:** 2024-07-15

**Authors:** Chuanmao Zheng, Jieqing Li, Honggao Liu, Yuanzhong Wang

**Affiliations:** a College of Agronomy and Biotechnology, Yunnan Agricultural University, Kunming, 650201, China; b Medicinal Plants Research Institute, Yunnan, Academy of Agricultural Sciences, Kunming 650200, China; c Yunnan Key Laboratory of Gastrodia and Fungi Symbiotic Biology, Zhaotong University, Zhaotong 657000, Yunnan, China

**Keywords:** Boletes, Amino acid metabolomics, LC-MS, FT-NIR, ATR-FTIR, 2DCOS

## Abstract

The taste and aroma of edible mushrooms, which is a criterion of judgment for consumer purchases, are influenced by amino acids and their metabolites. Sixty-eight amino acids and their metabolites were identified using liquid chromatography mass spectrometry (LC-MS), and 16 critical marker components were screened. The chemical composition of different species of boletes was characterized by two-dimensional correlation spectroscopy (2DCOS) to determine the sequence of molecular vibrations or group changes. Identification of boletes species based on partial least squares discrimination (PLS-DA) combined with Fourier transform near-infrared spectroscopy (FT-NIR) and Fourier transform infrared spectroscopy (ATR-FTIR), residual convolutional neural network (ResNet) combined with three-dimensional correlation spectroscopy (3DCOS) was performed with 100% accuracy. Partial least squares regression (PLSR) analysis showed that FT-NIR and ATR-FTIR spectra were highly correlated with the amino acids and their metabolites detected by LC-MS. All models had achieved an R^2^p of 0.911 and an RPD >3.0. The results show that FT-NIR and ATR-FTIR spectroscopy in combination with chemometrics methods can be used for rapid species identification and estimation of amino acids and their metabolites content in boletes. This study provides new techniques and ideas for the authenticity of species information and the quality assessment of boletes.

## Highlights

• Boletes are characterized by FT-NIR, ATR-FTIR, LC-MS, and 2DCOS.
• PLS-DA and ResNet correctly categorize boletes by species.
• An accurate quality prediction model was built through PLSR analysis.
• FT-NIR and ATR-FTIR spectra were feasible for estimating the quality of boletes.

## Graphical abstract

Graphical abstract

**Figure:**
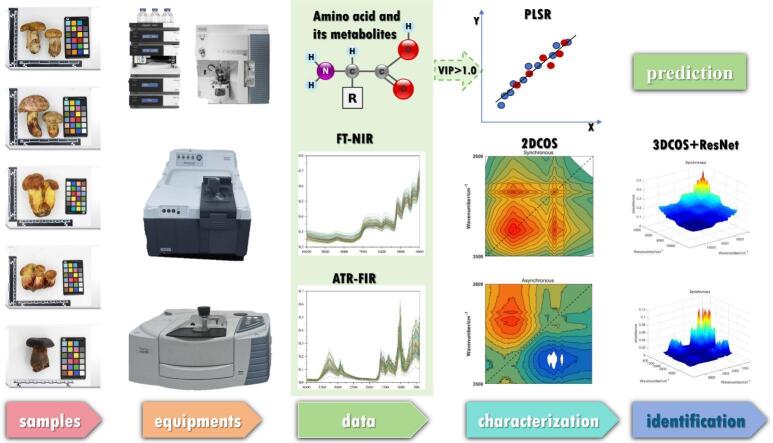

## 1 Introduction

While the population continues to grow, the land area shrinks and food resources are challenged, people are more interested in consuming natural, green and sustainable foods. Wild edible mushrooms are a good source of protein, dietary fiber, vitamins, minerals, and phenolic compounds that have a distinctive flavor and are widely preferred by consumers (Marçal et al., 2021). The protein content of wild-edible mushrooms is comparable to that of meat and eggs and much higher than that of vegetables and cereals (Zhang et al., 2018). There are many wild edible mushrooms, among which boletes are widely loved for their flavor (Yan, Liu, Zhang, et al., 2022). It is abundant in amino acids, which are important for human health, including nine essential amino acids (Zheng et al., 2023b). Furthermore, amino acids contribute to aroma and flavor and can be classified as sweet, fresh, and bitter according to their organoleptic properties (Hou et al., 2024; Jia et al., 2022). Lysine, proline, and methionine are involved in non-enzymatic browning, such as the Maillard reaction (Park et al., 2024).

With the increase in consumer demand, the quality and safety of wild edible mushrooms have become a growing concern (Yan, Liu, Li, et al., 2022). Their interspecies morphological similarity is high, and poisoning incidents caused by mixing poisonous mushrooms and edible mushrooms for sale have occasionally occurred. Therefore, the accurate identification of edible mushroom species is a prerequisite and foundation for consumer safety, in-depth development and research, and market quality control. Rapid predictive analysis of amino acids and their metabolites in edible mushrooms can help with their quality evaluation, health product development, aroma formation, and investigation of chemical reaction mechanisms.

Today, the main methods for analyzing the chemical composition of matrices include automatic amino acid analysis, high-performance liquid chromatography (HPLC), gas chromatography–mass spectrometry (GC–MS), and liquid chromatography-mass spectrometry (LC-MS) (Lv et al., 2023; Yin et al., 2022). These chemical detection methods have the disadvantages of being time-consuming, labor-intensive, and potentially harmful to human health and the environment. Traditional methods of species identification that rely on morphological and molecular identification are time-consuming when faced with large sample sizes. Currently, spectroscopic techniques have great potential for qualitative and quantitative analysis of food products, which are faster and easier (Serna-Escolano et al., 2024). Spectroscopic techniques reflect differences in the internal chemical composition of an object. The principle is the formation of absorption peaks by vibrational leaps of chemical bonds or chemical groups. (Zhang & Wang, 2023). Nevertheless, previous reports have only characterized the possible chemical compositional differences of different samples based on the positions and intensities of the absorption peaks of one-dimensional spectra. One-dimensional spectra suffer from the defect of overlapping chemical information and require cumbersome preprocessing methods to enhance the inter-spectral variability. Two-dimensional correlation spectroscopy images (2DCOS) allow a gradual increase in resolution and precise characterization of chemical bonds or groups without preprocessing (Lin et al., 2023). The residual convolutional neural network (ResNet) model is the superior image processing technique that overcomes the drawbacks of gradient descent in the Convolutional neural network (CNN) model, and can be combined with 2DCOS images to discriminate the samples in terms of species, origin, etc. (Chen et al., 2022; Li et al., 2022; Yan, Liu, Li et al., 2022; Yan, Liu, Zhang, et al., 2022). Recently, three-dimensional correlation spectral images (3DCOS) have enhanced the spectral information based on 2DCOS (Dong et al., 2023). The usability of 3DCOS combined with ResNet models was also confirmed in another study, where Liu et al. (2023) applied FT-MIR to successfully distinguish between wild and cultivated *Gastrodia elata*.

Partial least squares regression (PLSR), with strong covariance, correlation, and noisy data handling, is one of the most commonly used methods for predicting quality traits from infrared spectroscopy. Yan et al. (2023) used Fourier transform near-infrared spectroscopy (FT-NIR) in conjunction with PLSR for rapid prediction of nucleoside content in Boletes. Polysaccharides and moisture in *Ganoderma lucidum* were non-destructively detected by NIR spectroscopy and machine learning algorithms(Ni et al., 2023). Rapid quantification of adenosine content in commercially available boletes has been achieved using the FT-NIR technique combined with the PLSR model (Deng et al., 2024).

Hitherto, no study has been found on the rapid estimation of amino acids and their metabolites in boletes for the quality assessment using infrared spectroscopy. Consequently, the objectives of this study were to: i) demonstrate the feasibility of 2DCOS to characterize differences and variations in the chemical composition of different species; ii) assess the ability of FT-NIR (10,000–4000 cm^−1^) and ATR-FTIR (4000–400 cm^−1^) to discriminate between bolete species; iii) explore the potential of FT-NIR and ATR-FTIR for the prediction of amino acids in the nutritional quality of boletes.

## 2 Materials and methods

### 2.1 Sample collection

**Fig. 1:**
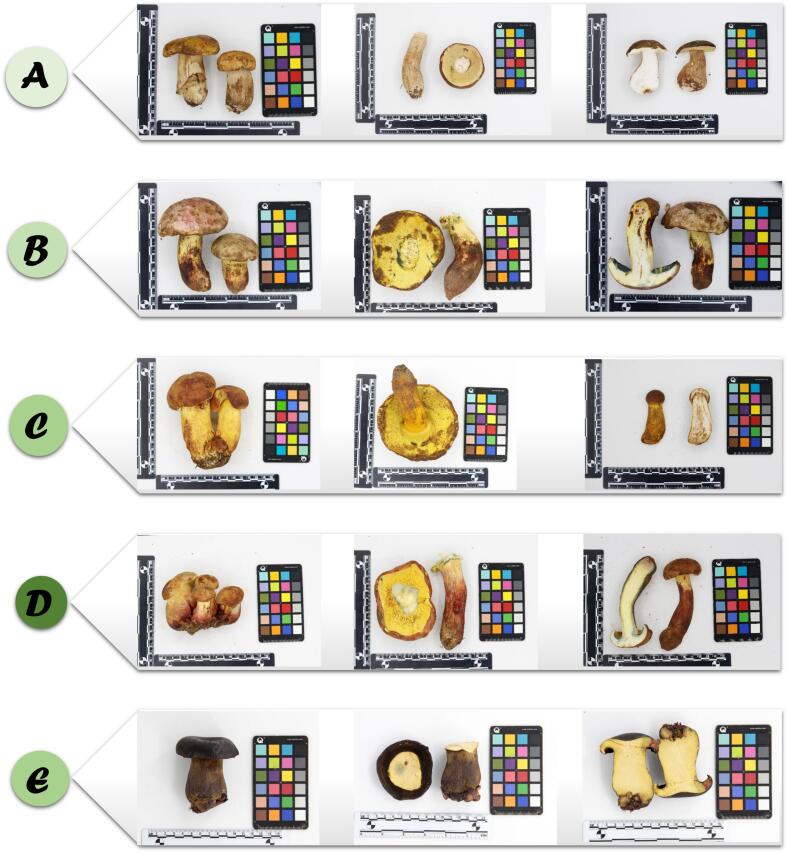
Morphological characteristics of the samples. (A) *Boletus bainiugan*, (B) *Butyriboletus roseoflavus*, (C) *Rugiboletus extremiorientalis*, (D) *Lanmaoa asiatica*, (E) *Phlebopus portentosus*.

Five species of mushrooms, specifically *Boletus bainiugan*, *Butyriboletus roseoflavus*, *Rugiboletus extremiorientalis*, *Lanmaoa asiatica*, and *Phlebopus portentosus*, were collected from Yunnan, China. Relevant information can be found in Table S1. Their morphological characteristics are shown in Fig. 1. It is worth mentioning that *Phlebopus portentosus* is a type of cultivated mushroom, while the rest of the mushrooms were gathered from the field. All mushrooms were dried in a hot air drying oven at 55 °C until they reached a constant weight. After pulverizing the samples, the resulting powder, which had passed through a 100 mesh sieve, was sealed and stored. (See Table 1.)

**Table 1:** Content of 16 important markers of amino acids and their derivatives in different boletes species (X ± *SD*).

Compounds	*Boletus bainiugan*	*Butyriboletus roseoflavus*	*Rugiboletus extremiorientalis*	*Lanmaoa asiatica*	*Phlebopus portentosus*
1-Methylhistidine (mg/kg)	12.64 ± 0.54^b^	21.11 ± 1.78^a^	4.41 ± 0.10^d^	6.69 ± 0.45^d^	6.87 ± 0.47^d^
3-N-Methyl-L-Histidine (mg/kg)	2.99 ± 0.14^c^	156.09 ± 15.44^a^	9.43 ± 0.44^c^	48.55 ± 3.55^b^	6.99 ± 1.01^c^
Succinic Acid (mg/kg)	16.87 ± 1.77^d^	7.28 ± 0.42^e^	23.93 ± 0.35^b^	20.89 ± 0.47^c^	28.37 ± 2.25^a^
L-Asparagine Anhydrous (g/kg)	0.94 ± 0.04^a^	0.52 ± 0.03^c^	0.94 ± 0.02^a^	0.78 ± 0.04^b^	0.50 ± 0.08^c^
Trimethylamine N-Oxide (mg/kg)	0.31 ± 0.02^b^	0.10 ± 0.04^c^	0.01 ± 0.009^d^	0.05 ± 0.004^d^	0.52 ± 0.07^a^
N-Acetylaspartate (g/kg)	0.10 ± 0.01^b^	0.15 ± 0.01^a^	0.05 ± 0.002^d^	0.05 ± 0.005^cd^	0.07 ± 0.02^c^
Urea (mg/kg)	5.30 ± 0.40^d^	9.13 ± 1.25^a^	56.74 ± 2.56^a^	6.04 ± 0.50^b^	11.88 ± 2.46^d^
L-Pipecolic Acid (mg/kg)	15.03 ± 2.20^b^	1.20 ± 0.03^d^	1.53 ± 0.14^d^	4.10 ± 0.16^c^	18.68 ± 2.32^a^
3-Iodo-L-Tyrosine (mg/kg)	0.37 ± 0.02^d^	0.12 ± 0.01^e^	0.74 ± 0.08^b^	0.48 ± 0.04^c^	1.78 ± 0.09^a^
(5-L-Glutamyl)-L-Alanine (mg/kg)	65.96 ± 5.87^c^	34.45 ± 2.86^d^	5.17 ± 0.32^e^	127.94 ± 5.87^a^	75.32 ± 5.77^b^
L-Ornithine (g/kg)	3.52 ± 0.30^b^	4.19 ± 0.18^a^	1.33 ± 0.28^d^	2.76 ± 0.22^c^	1.31 ± 0.26^d^
Glutathione Oxidized (g/kg)	3.31 ± 0.26^a^	0.92 ± 0.12^c^	1.55 ± 0.16^b^	0.82 ± 0.05^c^	0.15 ± 0.03^d^
N-Isovaleroylglycine (mg/kg)	0.13 ± 0.02^d^	0.39 ± 0.09^a^	0.16 ± 0.02^cd^	0.27 ± 0.01^b^	0.23 ± 0.08b^c^
Nα-Acetyl-l-glutamine (mg/kg)	180.71 ± 55.63^a^	71.77 ± 8.81^d^	123.52 ± 7.71^b^	89.94 ± 2.42^c^	128.83 ± 5.61^d^
L-Arginine (g/kg)	0.71 ± 0.04^b^	0.27 ± 0.05^d^	0.05 ± 0.007^e^	0.94 ± 00.02^a^	0.47 ± 0.05^c^
l-Glutamine (g/kg)	6.00 ± 0.134^c^	5.00 ± 0.08^d^	6.69 ± 0.12^b^	8.95 ± 0.39^a^	5.01 ± 0.29^d^

Note: Means with different letters within a column are significantly different (P < 0.05).

### 2.2 Amino acids and their metabolites

The amino acid and its metabolites were detected by MetWare (http://www.metware.cn/) based on the AB Sciex QTRAP 6500 LC-MS/MS platform.

#### 2.2.1 Chemicals and reagents

HPLC-grade acetonitrile (ACN) and methanol (MeOH) (Merck, located in Darmstadt, Germany). MilliQ water (Millipore in Bradford, USA). Standards, ammonium acetate and formic acid (Sigma-Aldrich, located in St. Louis, MO, USA). We used concentration of 1 mg / mL in MeOH to prepare the standard stock solutions and stored them at −20 °C. For analysis, we created working solutions by diluting the stock solutions with MeOH. Information on amino acid standards is detailed in Table S2.

#### 2.2.2 Sample preprocessing

After smashing the sample, 0.05 g was mixed with 500 μL of 70% methanol/water and then centrifuged for 3 min at 2355 ×*g* centripetal force. The mixture was centrifuged for 10 min at 11304 ×*g* centripetal force at 4 °C. The resulting supernatant was then placed in a − 20 °C refrigerator for 30 min and centrifuged again for 10 min at 11304 ×*g* centripetal force at 4 °C. Finally, 200 μL of the supernatant was transferred through the Protein Precipitation Plate for LC-MS analysis.

#### 2.2.3 UPLC conditions

The sample extracts were analyzed using the UPLC ExionLC AD and QTRAP® 6500+ System (https://sciex.com/). Analytical conditions were as follows: HPLC column, ACQUITY BEH Amide (1.7 μm, 100 mm × 2.1 mm i.d.); solvent system, water and acetonitrile with 2 mM ammonium acetate and 0.04% formic acid; flow rate, 0.4 mL/min; temperature, 40 °C; injection volume: 2 μL. The total ion flow chromatogram is shown in Fig. S1.

#### 2.2.4 ESI-MS/MS

The AB 6500+ QTRAP® LC-MS/MS System has an ESI Turbo Ion-Spray interface that operates in both positive and negative ion modes. The ESI source has an ion source turbo spray and a source temperature of 550 °C. The ion spray voltage is 5500 *V* (Positive), and − 4500 V (Negative), and curtain gas (CUR) is set at 35.0 psi. A specific set of MRM transitions is monitored for each period on the basis of the eluted amino acid. The mass spectra of the amino acids and their metabolites are shown in Fig. S2.

### 2.3 FT-NIR spectroscopy

The Antaris II FT-NIR spectrometer from Thermo Fisher Scientific Inc. in the USA was used to collect FT-NIR spectra of all the samples. The spectral range was between 10,000–4000 cm^−1^, and diffuse reflectance mode was used. The spectral resolution was 8 cm^−1^, and each sample was acquired 64 times with two repetitions. The final value was calculated by averaging the results. The background was collected every 64 min. The measurements were taken in a dry atmosphere at 25 °C. Raw FT-NIR spectroscopy image is shown in Fig. S3.

### 2.4 ATR-FTIR spectroscopy

The FTIR spectrometer from Thermo Fisher Scientific in the USA was used in the experiment. It was equipped with a single-point diamond attenuated total reflection (ATR) accessory and OMNIC 9.77 software. To remove the effects of atmospheric H_2_O and CO_2_, a pre-acquisition background was taken at 30 min intervals. For spectral acquisition, two grams of the sample were placed on the ATR and acquired over a range of 4000–400 cm^−1^. The scans were set at a resolution of 8 cm^−1^, with 16 spectral scans per set. To obtain an average infrared spectrum, the scans were repeated three times. Before each acquisition, the ATR was wiped with ethanol (75%). This experiment was carried out at room temperature. Raw ATR-FTIR spectroscopy image is shown in Fig. S3.

### 2.5 Correlation spectral image acquisition

The 2DCOS algorithm was used to compute the two-dimensional correlation spectrum based on Noda's theory (Noda, 2014). In Eq. (1), the variable *t* represents the range of values in the perturbation interval. The variable *n* represents the number of spectral measurement steps within the perturbation interval. The dynamic spectral intensity of the υ-variable is represented by the column vector *k* (Dong et al., 2023).

(1) $$k\left(\upsilon \right)=\left\{\begin{array}{c} k\left(\upsilon ,t_{1}\right)\\ k\left(\upsilon ,t_{2}\right)\\ k\left(\upsilon ,t_{n}\right) \end{array}\right)$$

The two-dimensional association strengths of υ1 and υ2 are denoted as (Eqs. (2), (3)):

(2) $$\Phi \left(\upsilon 1,\upsilon 2\right)=\frac{1}{m-1}{k_{\left(\upsilon 1\right)}}^{T}∙k_{\left(\upsilon 2\right)}$$

(3) $$\varphi \left(\upsilon 1,\upsilon 2\right)=\frac{1}{m-1}{k_{\left(\upsilon 1\right)}}^{T}∙Ν∙k_{\left(\upsilon 2\right)}$$

where Φ and φ are the two-dimensional synchronous and asynchronous correlation strengths, respectively, and N is the Hilbert-Noda matrix, as defined by Eq. (4):

(4) $$N_{\mathrm{j\gamma}}=\left\{\begin{array}{c} 0\;j=0\\ \frac{1}{\pi \left(\gamma -1\right)}\;j\neq 0 \end{array}\right)$$

The matlab (2023a) can generate synchronous and asynchronous 2DCOS spectra by using Eqs. (2), (3) respectively. Synchronous and asynchronous 3DCOS are generated using the surf function for image display, similar to 2DCOS. To generate 2DCOS and 3DCOS images, two variables are required. The first variable is the average spectrum, while the second variable is the *i*-th spectrum. To categorize the data into train set (2/3), test set (1/3), the Kennard-Stone (K—S) algorithm was used. We saved the spectra as JPEG images with a consistent size of 128 × 128 pixels.

### 2.6 Chemometrics

The quality differences between various species of boletes were determined using multivariate statistical analysis. Initially, independent variables, such as amino acid and their derivatives content based on LC-MS, peak intensities based on ATR-FTIR and FT-NIR spectroscopy were identified, and dependent variables, i.e., different species, were determined.

We utilized different software for their data analysis. To perform the principal component analysis (PCA) and the partial least squares discriminant analysis (PLS-DA), we used SIMCA 14.1 and OriginPro 9.0 software. Meanwhile, Unscrambler X10.4 was the software of choice for PLSR analysis. For ResNet modeling, we used Spyder (Anaconda3).

The K—S algorithm selected 2/3 of the dataset as the train set and 1/3 as the test set. On the basis of this, a PLS-DA model was built and cross-validated 7-fold. The performance of the model was evaluated using several metrics, including the fit correlation coefficient (R^2^), the cross-validation correlation coefficient (Q^2^), the cross-validation root mean square error (RMSECV), and the root mean square error of prediction (RMSEP). The model is considered stable when RMSECV and RMSEP are close to 0; R^2^ and Q^2^ are close to 1. Finally, the permutation test was used to verify the risk of overfitting the model. The model was evaluated using several parameters, including the coefficient of determination (R^2^c), the prediction coefficient of determination (R^2^p), the corrected root mean square error (RMSEC), RMSEP, and the ratio of performance to deviation (RPD). The predictive performance of the model is assessed by RPD, which is calculated using Eq. (5). An RPD value of <1.4 means that the model is not very accurate, while an RPD value between 1.4 and 3.0 is good enough to use for content prediction. If the RPD value is >3.0, the model is highly accurate and can be used for practical detection (Tian, Chen, Gui, et al., 2021; Tian, Chen, Zhang, et al., 2021).

(5) $$\mathrm{RPD}=\frac{\mathrm{SD}}{\text{RMSEP}}$$

where *SD* is the standard deviation. To determine the significance of the differences in the experimental data, IBM SPSS Statistics 25 was used to perform ANOVA and Duncan's multiple range test (*P* < 0.05).

Based on 3DCOS images, a 12-layer ResNet model is built with a weight decay coefficient λ of 0.0001 and a learning rate of 0.01. It contains identity blocks and convolutional blocks, which ensure that the dimensionality of the output result is the same as the input result. When the dimensions of the input and output are consistent, the identity block is considered and vice versa, the convolution block is considered. The working principle of the ResNet model is shown in Fig. S4: the input data are normalized by the convolutional layer and Batchnorm layer respectively; the Relu function carries out the nonlinear activation; the features are extracted by entering the Identify block, the convolutional block, and the global average pooling layer; the flat layer downsizes the multidimensional features, and the fully connected layer outputs the results. Model performance is evaluated based on train set accuracy, test set accuracy, and loss value.

## 3 Results and discussion

### 3.1 Liquid chromatography mass spectrometry

**Fig. 2:**
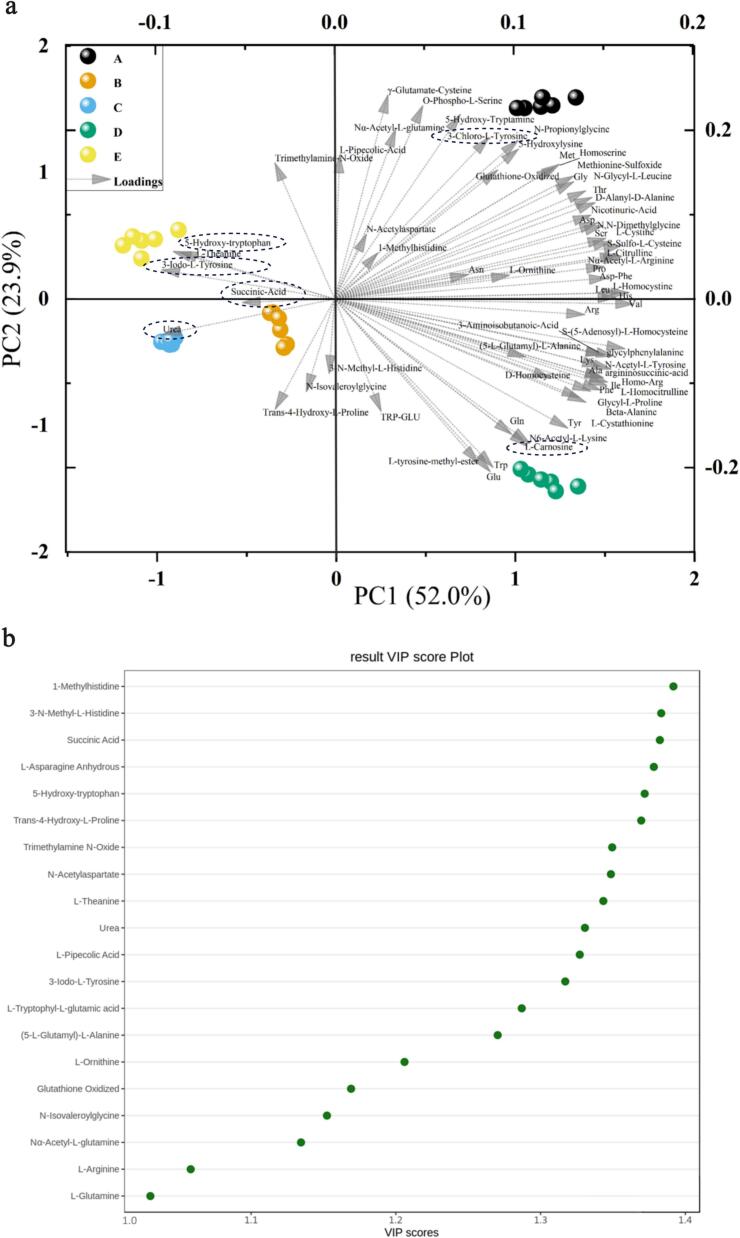
(a) Dual-label plot of PCA based on amino acids and their metabolites determined 92.6% of explained variance, (b) plot of VIP scores for amino acids and their metabolites with VIP > 1.0. (A) *Boletus bainiugan*, (B) *Butyriboletus roseoflavus*, (C) *Rugiboletus extremiorientalis*, (D) *Lanmaoa asiatica*, (E) *Phlebopus portentosus*.

**Fig. 3:**
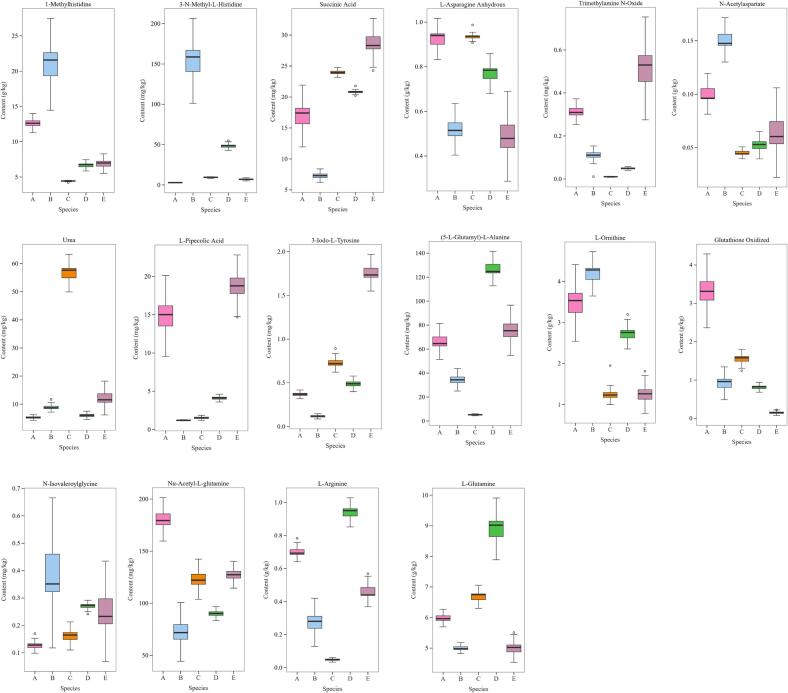
Boxplots of the 16 mass marker content of (A) *Boletus bainiugan*, (B) *Butyriboletus roseoflavus*, (C) *Rugiboletus extremiorientalis*, (D) *Lanmaoa asiatica*, (E) *Phlebopus portentosus*.

The taste of boletes is attributed to their chemical composition, where the role of amino acid metabolites is crucial. LC-MS analysis revealed that extracts from five different bolete species contained a total of 68 amino acids and their metabolites (Table S3). There are five species of boletes, including *Boletus bainiugan* 65, *Butyriboletus roseoflavus* 64, *Rugiboletus extremiorientalis* 61, *Lanmaoa asiatica* 64, and *Phlebopus portentosus* 63. Fig. 2a shows PCA double-labeled plots of amino acids and their metabolites generated by these five species of boletes. Variables with longer arrows are more critical in producing effects, with variables in the same direction showing a positive correlation, and samples close to the position of the variable's arrow having a strong correlation with that variable. Forexample, the location of Succinic Acid in the third quadrant favored the differentiation of *Butyriboletus roseoflavus* species from other species. Similarly, the presence of 5-Hydroxy-tryptophan and 3-Iodo-L-Tyrosine resulted in the clustering of *Phlebopus portentosus*. Furthermore, the presence of 3-Chloro-L-Tyrosine, l-Glutamine, and Urea played a significant role in the clustering of *Boletus bainiugan*, *Lanmaoa asiatica*, and *Rugiboletus extremiorientalis*, respectively. Generally, components with VIP scores (PLS-DA) >1.0 can be considered as significant feature components, with higher scores indicating greater significance (Fig. 2b) (He et al., 2023; Wang et al., 2021). PLS-DA identified 22 amino acids and their metabolites with VIP scores >1.0. Eventually, 16 amino acids and their metabolites were identified as important markers for predicting the quality of the five bolete species based on their shared composition. The study discovered that there were variations in the content among the five species of boletes. The 16 components of the critical markers were identified, as shown in Fig. 3. To minimize the variability in the data, the K—S duplex algorithm was used to divide the calibration set (20) and validation set (10). Using the sixteen significant quality markers, a PLSR model was built using FT-NIR and ATR-FTIR spectroscopy. This model allows for swift analysis of boletes quality.

### 3.2 Profiling of spectral data for boletes

**Fig. 4:**
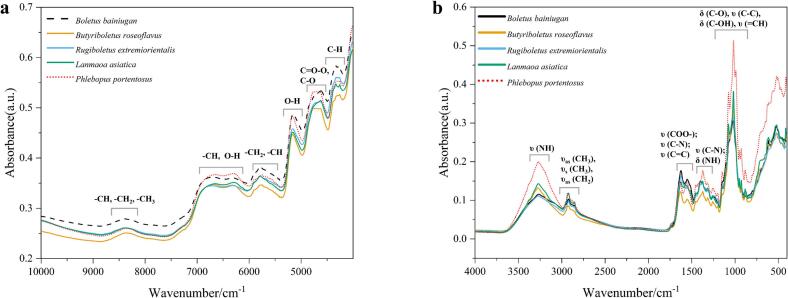
Average spectrograms of (a) FT-NIR and (b)ATR-FTIR.

FT-NIR and ATR-FTIR spectra were taken for *Boletus bainiugan*, *Lanmaoa asiatica*, *Rugiboletus extremiorientalis*, *Butyriboletus roseoflavus*, and *Phlebopus portentosus*. The spectral ranges were 10,000–4000 cm^−1^ and 4000–400 cm^−1^, respectively. The spectral profiles were mainly influenced by the chemical bonding of the chemical constituents, such as C—N, O—H, C—O, and C=O. Fig. 4 presents the average spectra of FT-NIR and ATR-FTIR of the different boletes. The spectra showed the same shape and position of the absorption peaks, but the intensity of the absorption varied among the samples. Fig. 4a shows the average FT-NIR spectra, indicating broader absorption peaks in the spectral band of 8516–8168 cm^−1^ with the highest point at 8350. This peak corresponds to the second overtone of functional groups CH, CH_2_, and CH_3_. In the spectral band of 7038–6024 cm^−1^, two consecutive broad peaks can be observed. These peaks can be attributed to higher protein and starch content and have maximums at 6776 cm^−1^ and 6286 cm^−1^, contributed by the second overtone of CH and OH functional groups, respectively. Additionally, peaks appear due to the first overtone of CH_2_ and CH functional groups near 5789 cm^−1^. Lastly, O—H and C—O stretching vibrations appear near 5160 cm^−1^ (Yu et al., 2022). The spectral range between 4828 and 4525 cm^−1^ is consistently broader and stronger in *Boletus bainiugan* and *Phlebopus portentosus*. This is due to the first overtone of the functional groups C=O-O, C—O, and OH. On the other hand, the spectral band between 4408 and 4188 cm^−1^ is likely to be related to proteins and lipids, with contributions from the second overtone of C—H and C-N-C (Drees et al., 2023; Foschi et al., 2024).

ATR-FTIR is a more informative method compared to FT-NIR. It provides detailed chemical information. The absorption peaks observed at 3269 cm^−1^ were larger and caused by the N—H stretching vibrations of protein peptides and amino acids. The spectral band of 2990–2849 cm^−1^ showed two absorption peaks, which were contributed by symmetric and asymmetric vibrations of fatty acid and polyol C—H. The peak shape and intensity of *Phlebopus portentosus* were the most significant. Distinctive peaks of amide I and amide II were detected between 1670 and 1550 cm^−1^ due to COO-, C=O, and C=C stretching vibrations in proteins, amino acids, and their derivatives. Among these, the most notable peak shapes and widths were observed in *Boletus bainiugan* (Martins et al., 2022). A major absorption peak and several additional peaks appeared in the 1470–1186 cm^−1^ spectral band, mainly contributed by the C-O-H, C—N stretching vibration, and N—H bending vibration modes of the amino acids (Lin et al., 2023). The most prominent absorption peak is found at 1018 cm^−1^ within the spectral band of 1186–864 cm^−1^. Additionally, several incidental peaks are attributed to the stretching vibration of C—C, =CH, and the bending vibration of C—O and C-OH of the polysaccharides (Lin et al., 2023; Tan et al., 2021). Additionally, areas where fingerprint signals were difficult to interpret, were located below 700 cm^−1^ (Foschi et al., 2024); they appeared to be distinguishing characteristics between cultivated and wild boletes, with *Phlebopus portentosus* being clearly distinguished from the other four wild boletes.

**Fig. 5:**
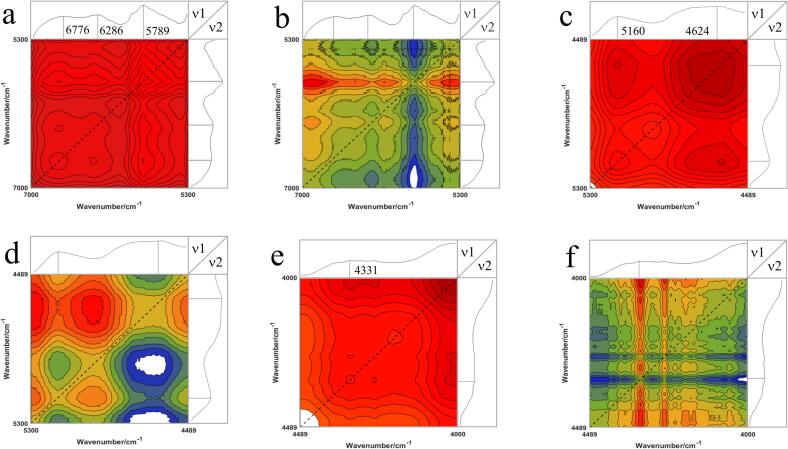
Synchronous and asynchronous two-dimensional correlation spectra (2DCOS)-FT-NIR correlation spectra of different boletes. (a) synchronous spectra in the 7000–5300 cm^−1^ region; (b) asynchronous spectra in the 7000–5300 cm^−1^ region; (c) synchronous spectra in the 5300–4489 cm^−1^ region; (d) asynchronous spectra in the 5300–4489 cm^−1^ region; (e) synchronous spectra in the 4489–4000 cm^−1^ region; and (f) 4489- asynchronous spectra in the 4000 cm^−1^ region.

Contour-based 2DCOS spectra were used to enhance resolution (red: positive absorption peak; blue: negative absorption peak). Auto-peaks, located at diagonal positions, indicate changes in spectral intensity between perturbed spectral pairs in different regions. The number and location of auto-peaks can be determined by analyzing the intensities and attributions of the peaks on the associated 1D spectrum. Cross peaks on non-diagonal lines suggest stronger interactions between spectral signals of different wavenumbers. FT-NIR was selected for three characteristic spectral bands for 2DCOS image analysis (Fig. 5). The 2DCOS-FT-NIR in the region of 7000–5300 cm^−1^ is shown in Fig. 5a and b. Two stronger auto peaks were observed at 6776 and 5789 cm^−1^, respectively, which are related to the vibration of C—H, and O—H chemical bonds. According to a study by An et al. (2024), if the cross peaks (*υ*1, *υ*2) in the synchronized spectra share the same sign as the cross peaks in the asynchronous spectra, then the high wave number changes occur first. However, if the signs do not match, then the low wave number changes occur initially. In this spectral band, the cross peaks in the synchronous spectra are all positive, while the asynchronous spectra have negative cross peaks, so the O—H at 5789 cm^−1^ starts to change first. Two auto peaks with stronger intensity were observed at 5160 cm^−1^ and 4624 cm^−1^ as shown in Fig. 5c. The cross peaks of the synchronous and asynchronous spectra did not coincide, as illustrated in Fig. 5d. The C—O vibration at 4624 cm^−1^ was observed to start. The spectral band at 4408–4188 cm^−1^ displays an auto peak at 4331 cm^−1^, which is associated with C—H in the protein. Both synchronous and asynchronous cross-peaks are positive, indicating that the C—H group changes first.

**Fig. 6:**
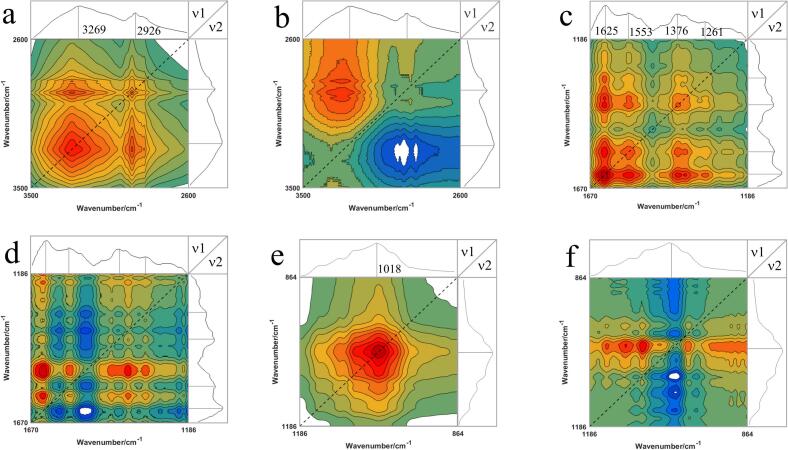
Synchronous and asynchronous two-dimensional correlation spectra (2DCOS)-ATR-FTIR correlation spectra of boletes. (a) synchronous spectra in the 3500–2600 cm^−1^ region; (b) asynchronous spectra in the 3500–2600 cm^−1^ region; (c) synchronous spectra in 1670–1186 cm^−1^ region; (d) asynchronous spectra in 1670–1186 cm^−1^ region; (e) synchronous spectra in the 1186–864 cm^−1^ region; (f) 1186–864 cm^−1^ region of the asynchronous spectrum.

Based on the occurrence of characteristic peaks, the ATR-FTIR spectrum was divided into three spectral bands: 3500–2600 cm^−1^, 1670–1186 cm^−1^, and 1186–864 cm^−1^ (Fig. 6). Fig. 6a illustrates a broad and strong auto peak at 3269 cm^−1^, related by the stretching vibration of N-H; another weak auto peak appears at 2926 cm^−1^. Its cross peaks are inconsistent in the synchronous and asynchronous spectra. So the C—H group at 2926 cm^−1^ starts to change first when the spectrum is formed. The result showed that there were three auto peaks detected at 1670 cm^−1^, 1553 cm^−1^, and 1376 cm^−1^. Additionally, all cross peaks in the synchronized spectra were positive as seen in Fig. 6c. Fig. 6d presents the 2DCOS for the range 1670–1186 cm^−1^ and it shows that the cross-peaks are positive, which agrees with the synchronized spectra. Therefore, the order of peak changes in the formation of the spectra is 1625 cm^−1^ → 1553 cm^−1^ → 1376 cm^−1^ → 1261 cm^−1^. In the 1186–864 cm^−1^ range, there is a strong auto peak at 1018 cm^−1^. This peak is associated with the stretching vibration of C—C, =CH, and the bending vibration of C—O, C-OH (Fig. 6e). The synchronous spectrum does not have any cross-peaks in this range. However, the asynchronous spectrum shows multiple cross-peaks in this range (Fig. 6f). These cross peaks are attributed to the interaction of 1018 cm^−1^ with the incidental absorption peaks.

### 3.3 Species identification analysis of boletes

#### 3.3.1 Exploratory analysis

**Fig. 7:**
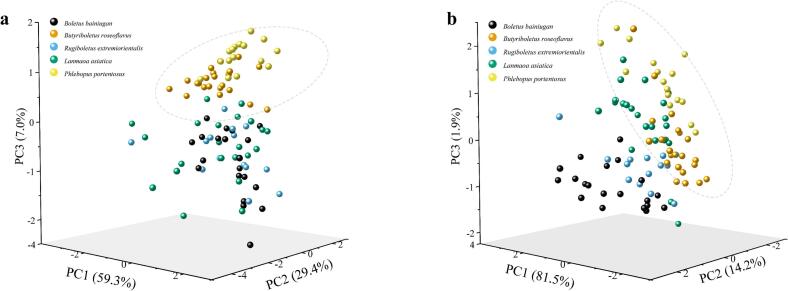
PCA score plots of (a) FT-NIR and (b) ATR-FTIR.

To assess the discriminatory power of bolete species, we used principal component analysis (PCA) to downscale spectral data obtained from FT-NIR and ATR-FTIR. The results of PCA based on FT-NIR spectra are shown in Fig. 7a, where the top three PCA scores accounted for 95.7% of the data. ATR-FTIR spectra contributed up to 97.6% of the PCA scores (Fig. 7b). It has been observed that *Butyriboletus roseoflavus* and *Phlebopus portentosus* tend to cluster together in the PCA results based on both spectra. This suggests that two species have similar chemical content and type, but are significantly different from other species. Nevertheless, both species overlap with each other and with other species, and their classification is difficult by PCA analysis.

#### 3.3.2 Partial least squares discriminant analysis

**Fig. 8:**
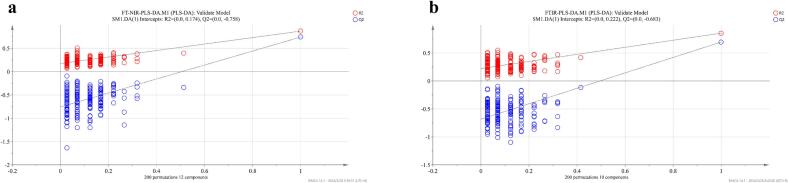
(a) FT-NIR and (b) ATR-FTIR to establish the 200 permutation test for PLS-DA.

To create species classification model for boletes, it's crucial to select a train set and test set that are generalized to all datasets and representative of all 5 species of boletes, as well as the variability of all data considered. The K—S algorithm was chosen to generate 62 samples for the train set and 33 for the test set. The study built PLS-DA and ResNet models to determine which techniques are most effective in distinguishing samples based on their species origin. The PLS-DA model built using FT-NIR spectral data was superior to the one built using ATR-FTIR spectral data. This was because FT-NIR had a lower root mean square error, and higher R^2^ (0.901) and Q^2^ (0.802) values (Table 2). As a whole, it was observed that the PLS-DA models built for both spectra had excellent performance, with species classified with 100% accuracy. The 200 permutation tests analyzed the possibility of model overfitting. The results showed that the true values on the right side were higher than the predicted values on the left side, and the R^2^ was <0.4, indicating that there was no overfitting of the model (Fig. 8) (Yan et al., 2023).

**Table 2:** PLS-DA modeling of five boletes species.

Spectral	LVs	RMSECV	R^2^	RMSEP	Q^2^	Train Acc (%)	Test Acc (%)
FT-NIR	12	0.206	0.901	0.130	0.802	100	100
ATR-FTIR	10	0.240	0.873	0.230	0.723	100	100

#### 3.3.3 Residual convolutional neural network models

**Fig. 9:**
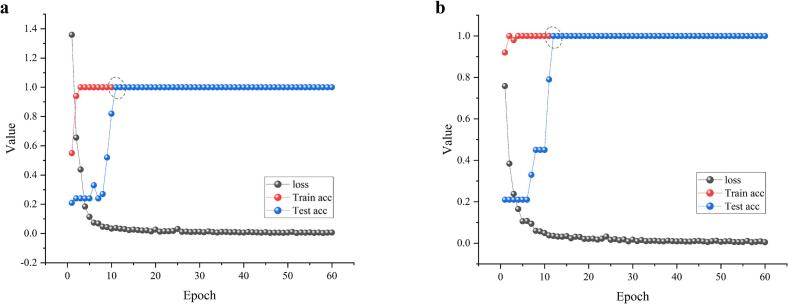
ResNet modeling results for (a) FT-NIR and (b) ATR-FTIR.

ResNet is a popular deep learning model that is often used for processing images. In Section 3.2, we parsed 2DCOS and extracted additional spectral information to increase the spatial dimension of the image. This resulted in the creation of a 3DCOS image, as illustrated in Fig. S5 (Li et al., 2023). An efficient and accurate species recognition model for boletes was established using ResNet based on 3DCOS. The performance of the model was evaluated using the cross-entropy loss function curve, the accuracy of the train set, and test set. The credibility and convergence ability of the model were proven since the cross-entropy loss function curve is close to 0 and the accuracy rate is close to 100% (Zheng et al., 2023a). Fig. 9 illustrates the ResNet modeling results based on 3DCOS images of FT-NIR (10,000–4000 cm^−1^) and ATR-FTIR spectra (4000–400 cm^−1^). It has been observed that in the case of FT-NIR, the accuracy of both train and test sets reaches 100% when the Epoch value reaches 11, with a loss value of 0.038. On the other hand, for ATR-FTIR, the accuracy also reaches 100%, but with an Epoch value of 12 and a loss value of 0.035. In general, the ResNet model exhibits reliable classification performance for boletes.

#### 3.3.4 Predictive analysis of the content of amino acids and derivatives

The present investigation used PLSR to investigate the correlation between FT-NIR, ATR-FTIR, and LC-MS data (Table 3). The contents of 16 quality markers (VIP > 1.0) were modeled with excellent PLSR models combined with FT-NIR and ATR-FTIR spectra. Both R^2^c and R^2^p were close to 1, with variation intervals of 0.884–0.997 and 0.911–0.996, respectively, while the RMSEP was close to 0, indicating a superior predictive ability of the model. Other important parameters to evaluate content prediction models include RPD, which has a value >3.0 indicating that the model has outstanding prediction accuracy (Huang et al., 2014). All RPD values exceeded 3.0, indicating excellent prediction performance and stability of amino acid quality marker content prediction models based on FT-NIR and ATR-FTIR spectroscopy. The maximum value of this amounted to 15.287, which is similar to the amino acid content predicted in *katsuobushi* by Park et al. (2024). The study concluded that FT-NIR and ATR-FTIR spectroscopy can accurately predict the metabolism of amino acids and their derivatives. The results suggest that the combination of infrared spectroscopy with chemometrics can be a valuable tool for quickly determining a variety of metabolites in boletes.

**Table 3:** PLSR modeling results for 16 quality markers.

		Calibration (*n* = 20)		Prediction (*n* = 10)		
Component	Spectral	R^2^_C_	RMSEC	R^2^_P_	RMSEP	RPD
1-Methylhistidine	FTIR	0.986	0.735	0.974	0.7141	6.070
	NIR	0.985	0.736	0.974	0.6835	6.959
3-N-Methyl-L-Histidine	FTIR	0.987	6.550	0.985	5.5600	7.241
	NIR	0.974	9.4020	0.986	5.5274	7.923
Succinic Acid	FTIR	0.974	1.168	0.985	1.1040	7.786
	NIR	0.967	1.3219	0.970	1.1195	5.669
L-Asparagine Anhydrous	FTIR	0.913	0.0008	0.962	0.0600	3.353
	NIR	0.895	0.0649	0.911	0.0600	3.295
Trimethylamine N-Oxide	FTIR	0.984	0.0241	0.984	0.0209	7.302
	NIR	0.987	0.0219	0.977	0.0229	4.883
N-Acetylaspartate	FTIR	0.914	0.0008	0.962	0.0060	6.739
	NIR	0.904	0.0122	0.979	0.0056	7.060
Urea	FTIR	0.959	4.0079	0.934	3.0943	4.977
	NIR	0.985	2.4175	0.964	2.1411	7.489
L-Pipecolic Acid	FTIR	0.992	0.6641	0.993	0.5078	8.504
	NIR	0.889	2.4801	0.955	1.3546	4.208
3-Iodo-L-Tyrosine	FTIR	0.992	0.0486	0.993	0.0403	11.468
	NIR	0.979	0.0838	0.983	0.0594	7.312
(5-L-Glutamyl)-L-Alanine	FTIR	0.997	2.0617	0.996	2.3424	15.287
	NIR	0.884	14.1278	0.978	6.7239	5.961
L-Ornithine	FTIR	0.979	0.1708	0.983	0.1721	7.726
	NIR	0.932	0.3078	0.974	0.1930	5.904
Glutathione Oxidized	FTIR	0.991	0.1034	0.987	0.0844	6.010
	NIR	0.969	0.1909	0.974	0.1579	5.910
N-Isovaleroylglycine	FTIR	0.992	0.0092	0.991	0.0064	10.159
	NIR	0.946	0.0244	0.947	0.0178	3.561
Nα-Acetyl-l-glutamine	FTIR	0.989	3.8332	0.996	2.6000	13.713
	NIR	0.978	5.5929	0.991	3.1356	10.704
L-Arginine	FTIR	0.985	0.0380	0.984	0.0354	7.701
	NIR	0.978	0.0471	0.985	0.0399	8.158
l-Glutamine	FTIR	0.914	0.4326	0.954	0.2580	4.617
	NIR	0.981	0.2041	0.983	0.1699	7.300

Note: R^2^c, coefficient of determination in calibration; R^2^p, coefficient of determination in prediction; RMSEC, root mean square error of calibration; RMSEP, root mean square error of prediction; RPD, residual predictive deviation.

## 4 Conclusions

The study aimed to quantitatively determine metabolites in different species of bolete extracts using FT-NIR and ATR-FTIR spectroscopic techniques along with chemometric methods. Amino acids and their derivatives were analyzed using LC-MS for five species of boletes. The findings revealed that the content and quantity of amino acid metabolites varied depending on the species of boletes. The vibrational information of chemical groups during the spectral formation of boletes was elucidated using 2DCOS image analysis to observe the sequence of absorption peak formation. The use of PCA, PLS-DA, and ResNet based on FT-NIR and ATR-FTIR spectra accurately classified the species, demonstrating the effectiveness of the two spectra for classification purposes. In the PLSR results, the FT-NIR and ATR-FTIR spectral data showed a high correlation with the LC-MS results, indicating that FT-NIR and ATR-FTIR spectroscopy combined with chemometrics is a suitable method for the quality evaluation of boletes. All models had achieved an R^2^p of 0.911 and an RPD >3.0. The chemical composition involved in this study is focused on quantitative data of amino acid metabolomics, and in the future, directions such as sensory testing and quantification of flavor components should be considered to comprehensively evaluate the quality of boletes.

## CRediT authorship contribution statement

**Chuanmao Zheng:** Writing – review & editing, Writing – original draft, Data curation, Conceptualization. **Jieqing Li:** Methodology, Investigation, Funding acquisition, Formal analysis. **Honggao Liu:** Software, Resources, Project administration, Funding acquisition. **Yuanzhong Wang:** Visualization, Validation, Software, Resources, Funding acquisition.

## Declaration of competing interest

The authors declare no known competing financial interests or relationships that could have appeared to influence the work reported in this paper.

## Data Availability

The data that has been used is confidential.

## Appendix A Supplementary data

Supplementary material Image 1
