# Supplementary material for: Application of ATR-FTIR and FT-NIR spectroscopy coupled with chemometrics for species identification and quality prediction of boletes
Source: Food Chem X. 2024 Jul 15;23:101661. doi: 10.1016/j.fochx.2024.101661 (PMC11304868; doi:10.1016/j.fochx.2024.101661)
Supplement: Supplementary file 1 — Supplementary material [file mmc1.docx]

**Supplementary Materials**

Application of ATR-FTIR and FT-NIR spectroscopy coupled with chemometrics for species identification and quality prediction of boletes

Chuanmao Zheng^a,b^, Jieqing Li^a^, Honggao Liu^c^^*^, and Yuanzhong Wang^b*^

^a^ College of Agronomy and Biotechnology, Yunnan Agricultural University, Kunming, 650201, China

^b^ Medicinal Plants Research Institute, Yunnan Academy of Agricultural Sciences, Kunming 650200, China

^c^ Yunnan Key Laboratory of Gastrodia and Fungi Symbiotic Biology, Zhaotong University, Zhaotong 657000, Yunnan China

^*^Corresponding author: Honggao Liu & Yuanzhong Wang

**Mr: Honggao Liu**, Yunnan Key Laboratory of Gastrodia and Fungi Symbiotic Biology, Zhaotong University, Zhaotong 657000, Yunnan China. E-mail: honggaoliu@126.com

**Mr: Yuanzhong Wang**, Medicinal Plants Research Institute, Yunnan Academy of Agricultural Sciences, Kunming 650200, China. E-mail: boletus@126.com


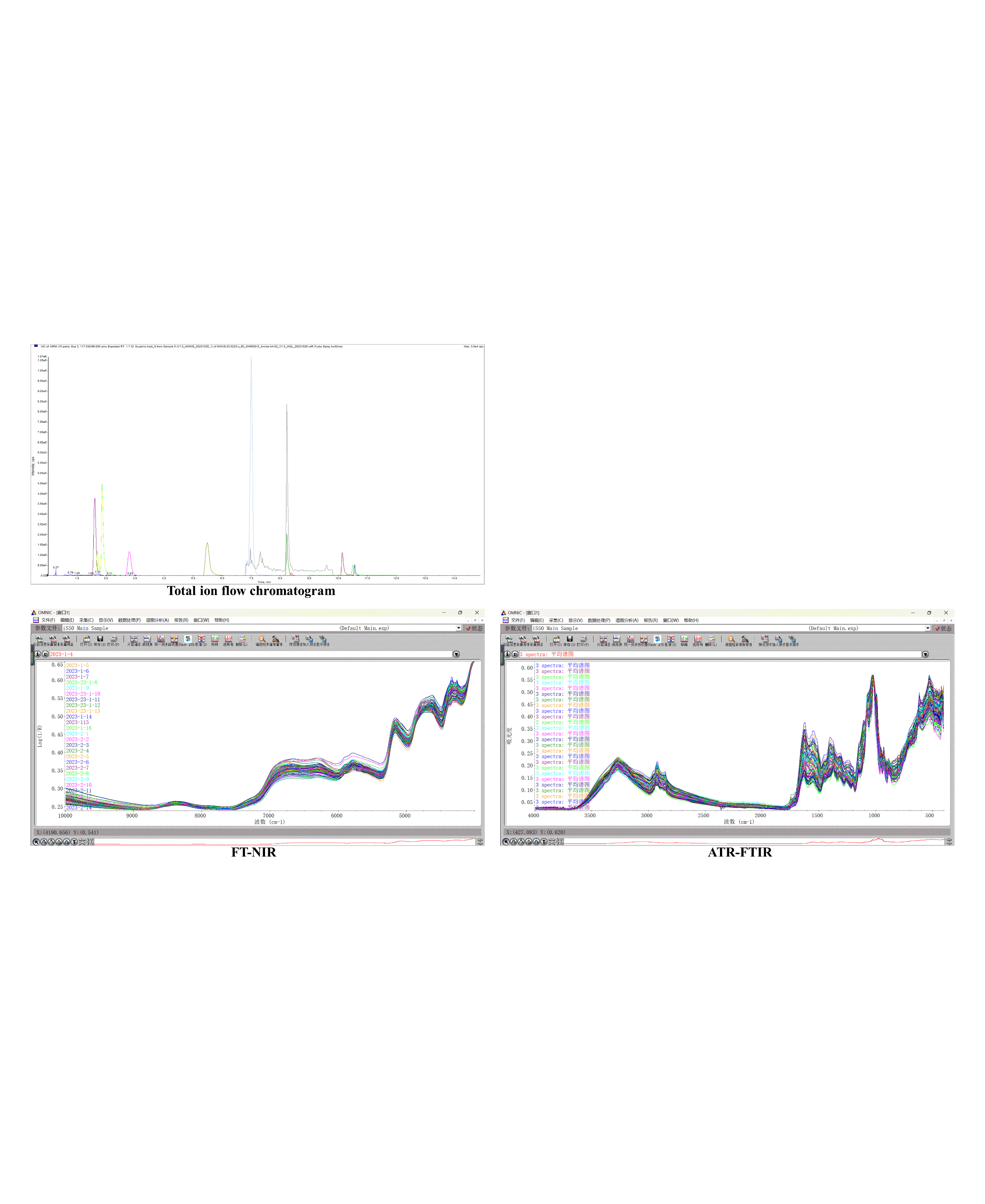


Figure S1. total ion flow chromatogram (TIC plot).


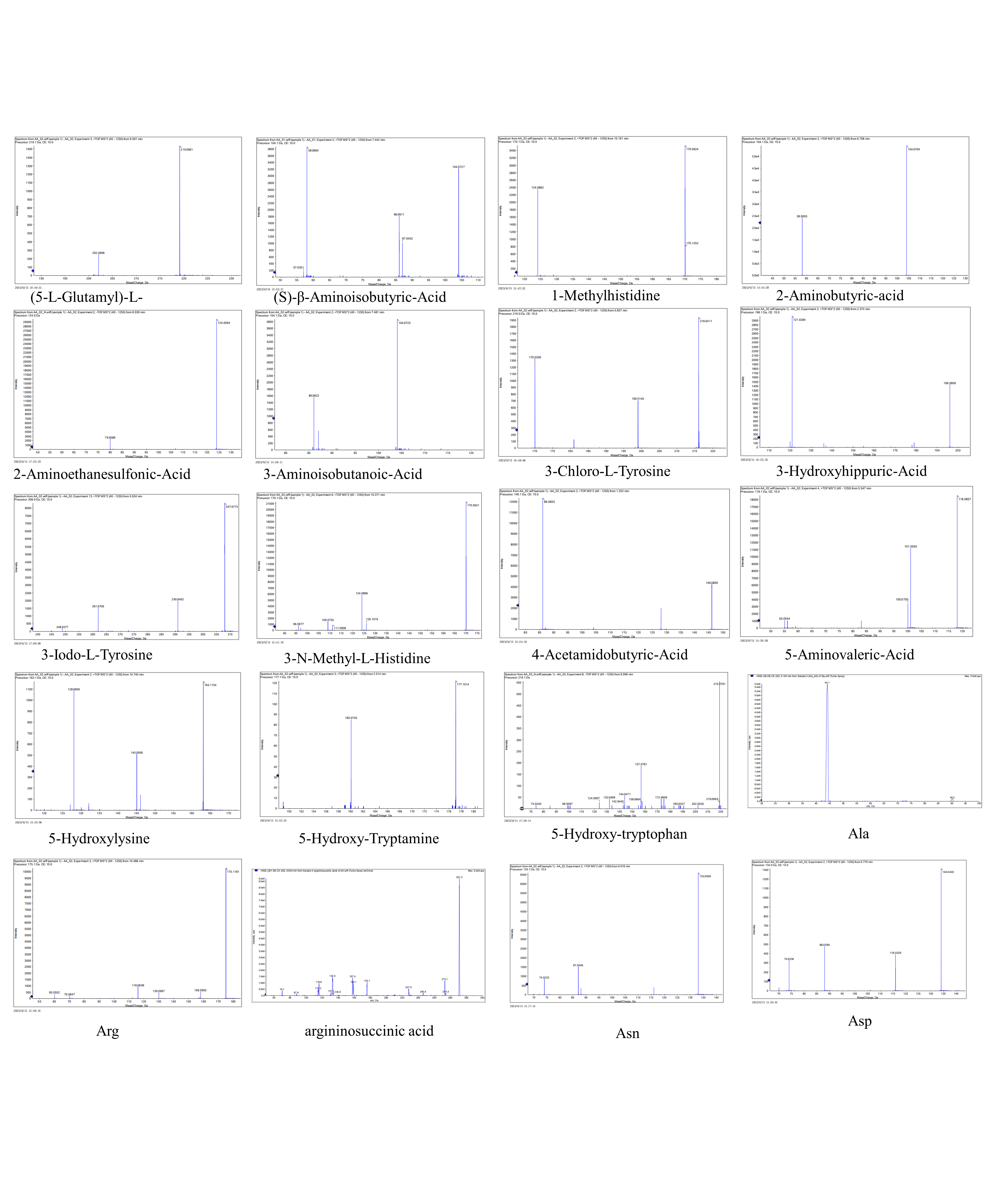


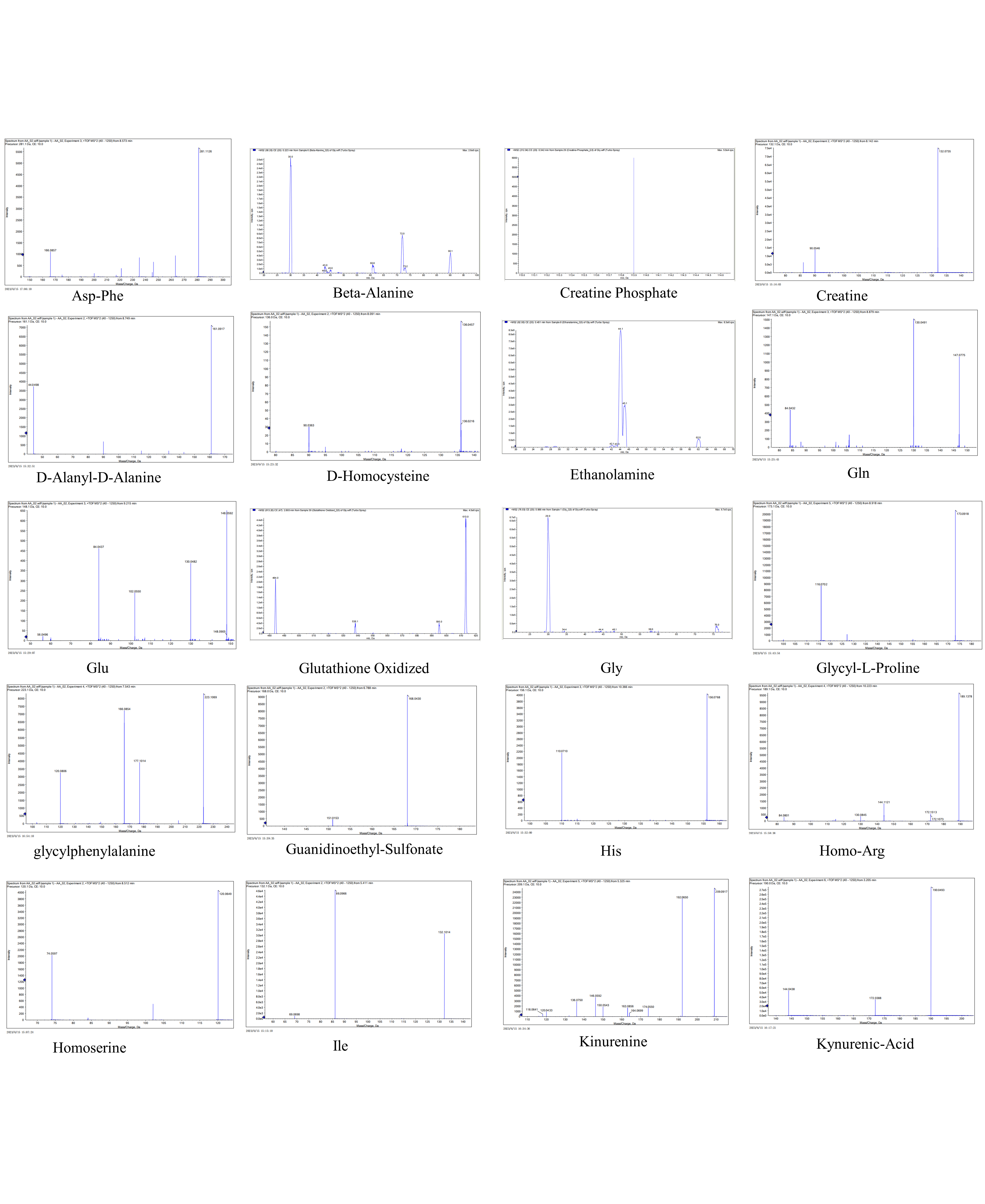


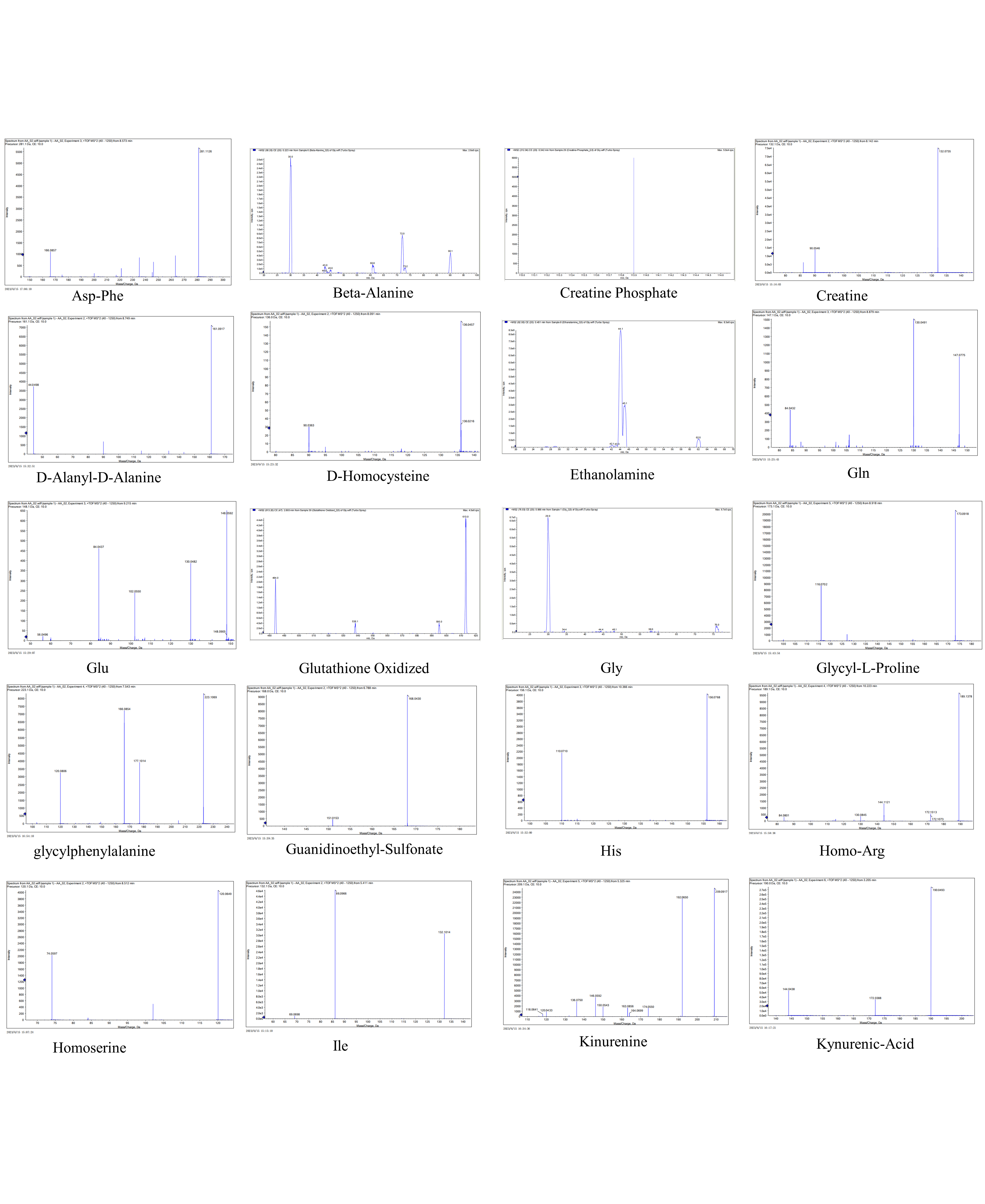


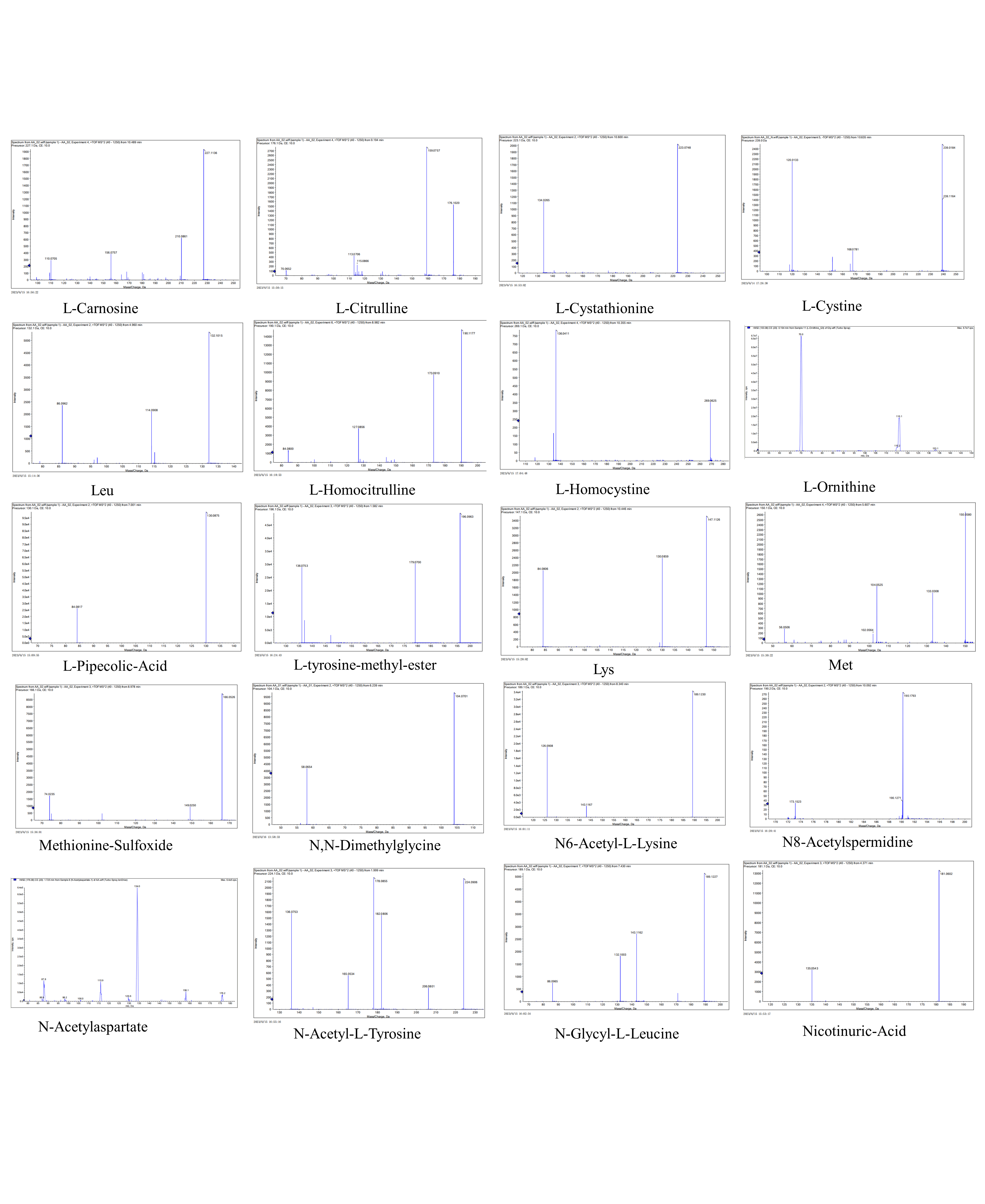


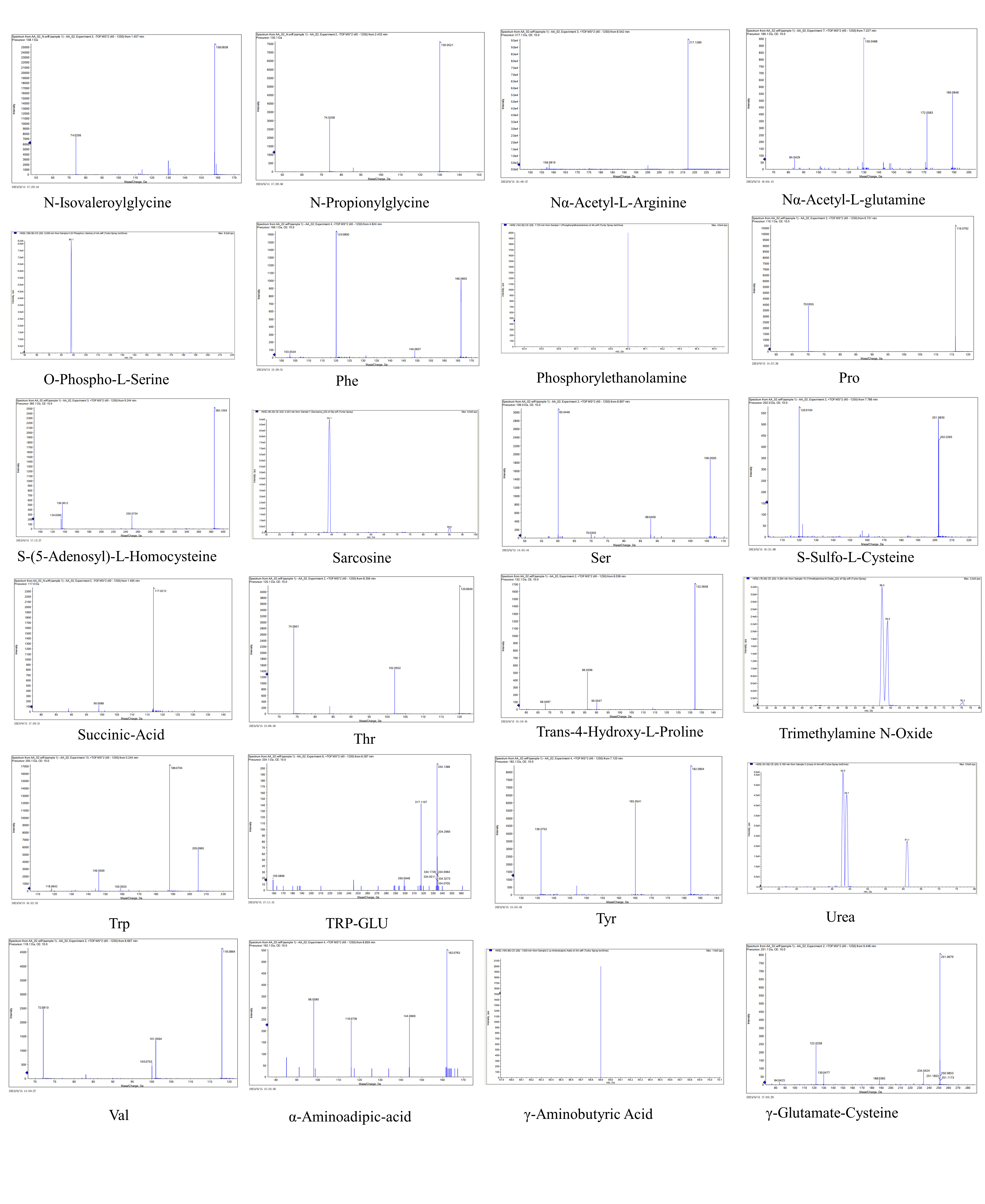


Figure S2. mass spectra of amino acids will their metabolites.


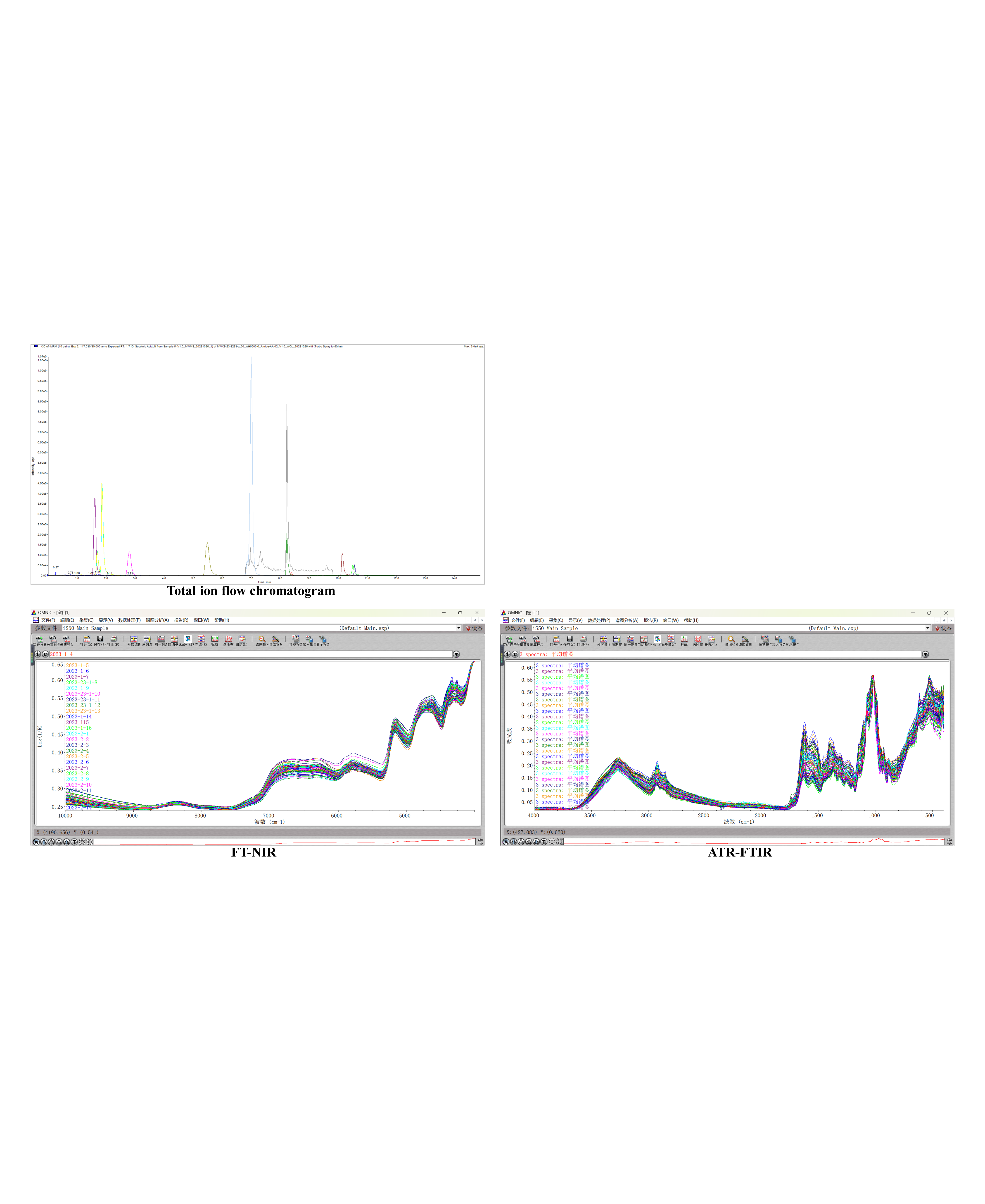


Figure S3. FT-NIR and ATR-FTIR raw spectrograms.


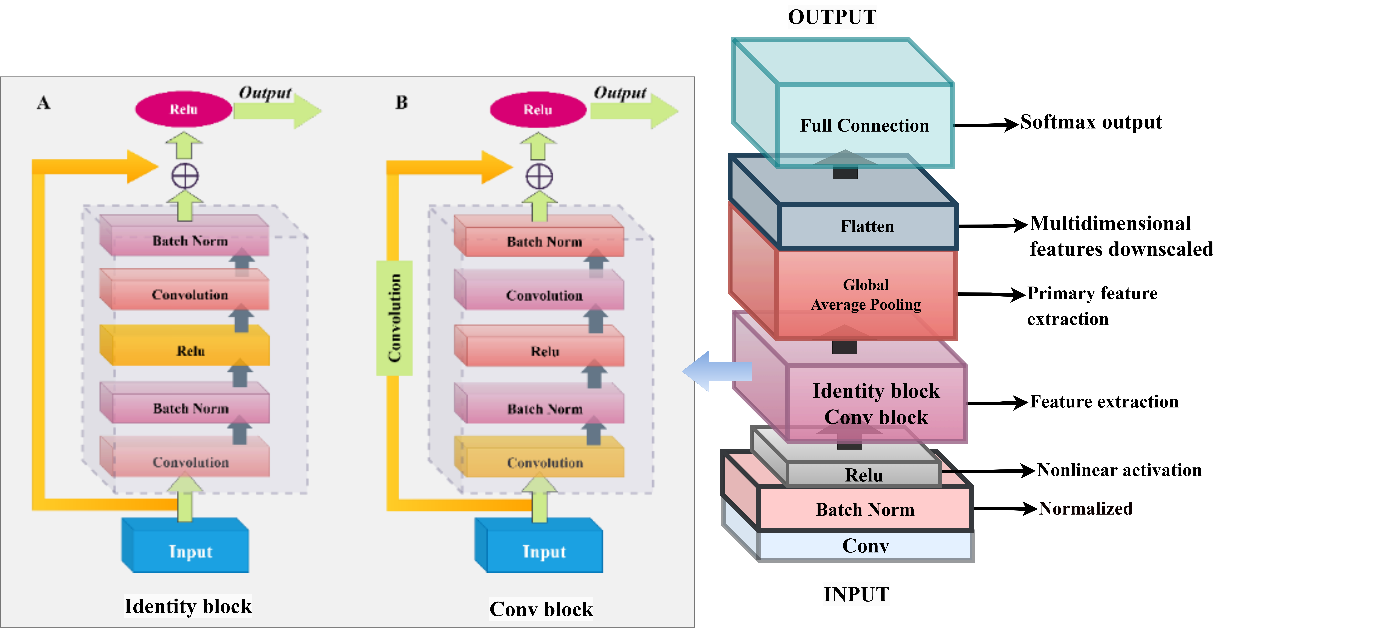


Figure S4 Structure of the residual convolutional neural network model.


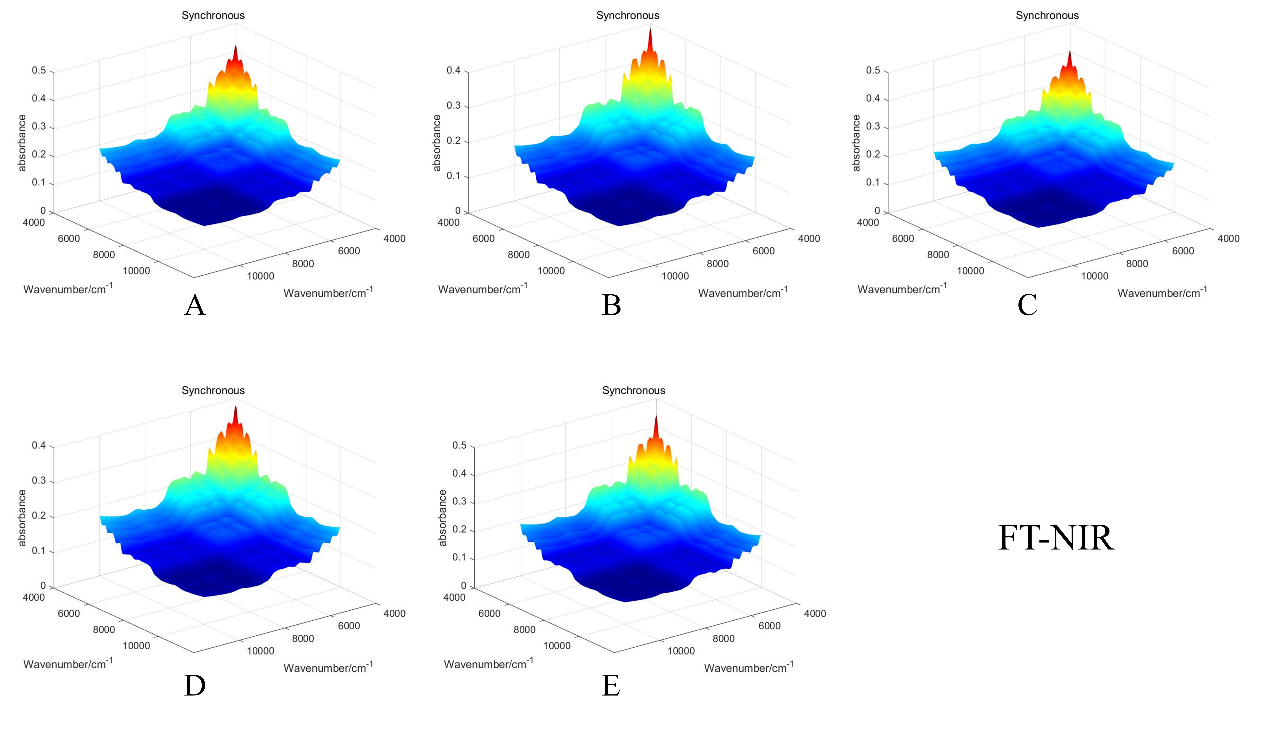


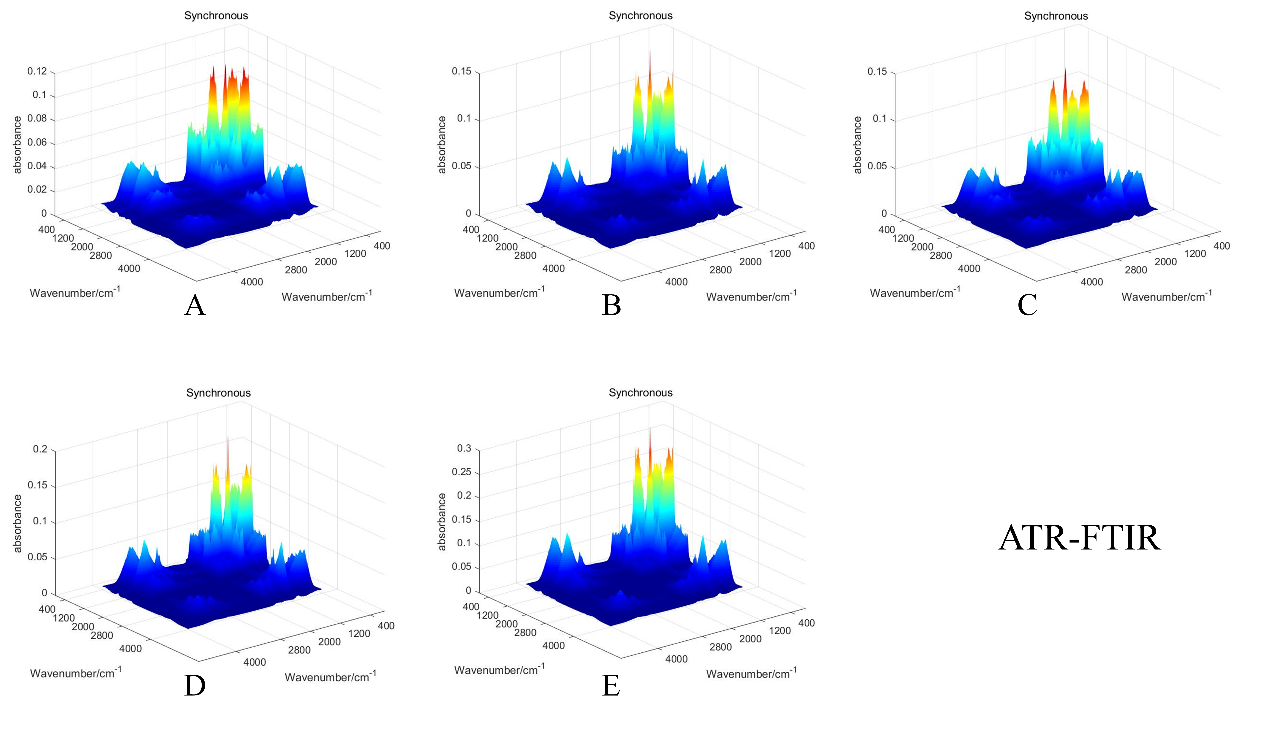


Figure S5 3DCOS image of (A) *Boletus bainiugan*, (B) *Butyriboletus roseoflavus*, (C) *Rugiboletus extremiorientalis*, (D) *Lanmaoa asiatica*, (E) *Phlebopus portentosus*.

Table S1 Sample information.

| Number | Species | Collecting site | Harvest date | longitudes (◦N) | Latitude (◦E) | Quantities | GC-MS number |
| --- | --- | --- | --- | --- | --- | --- | --- |
| A | *Boletus bainiugan* | Chedian Village, Wuding County, Chuxiong Prefecture, Yunnan Province, China. | 2023.7.12 | 102.40 | 25.53 | 20 | 1(1-3)^⁎^; 2(4-6); 3(7-9); 4(10-12); 5(13-15); 6(16-20) |
| B | *Butyriboletus roseoflavus* | Chedian Village, Wuding County, Chuxiong Prefecture, Yunnan Province, China. | 2023.7.12 | 102.40 | 25.53 | 23 | 1(1-3); 2(4-6); 3(7-9); 4(10-13); 5(14-17); 6(18-23) |
| C | *Rugiboletus extremiorientalis* | Fawo Village, Wuding County, Chuxiong Prefecture, Yunnan Province, China. | 2023.7.12 | 101.27 | 25.19 | 14 | 1(1-2); 2(3-4); 3(5-6); 4(7-8); 5(9-11); 6(12-14) |
| D | *Lanmaoa asiatica* | Lion Mountain, Wuding County, Chuxiong Prefecture, Yunnan Province, China. | 2023.7.12 | 102.37 | 25.54 | 22 | 1(1-3); 2(4-6); 3(7-9); 4(10-12); 5(13-16); 6(17-22) |
| E | *Phlebopus portentosus* | Xishuangbanna, Yunnan Province, China. | 2022.12.24 | 100.79 | 22.00 | 16 | 1(1-2); 2(3-4); 3(5-7); 4(8-10); 5(11-13); 6(14-16) |

^⁎^: Mixed samples corresponding to the sample label.

Table S2. Information on amino acid standards.

| Index | product number | brand | CAS number |
| --- | --- | --- | --- |
| L-Cystine | R014128-25g | Rhawn | 56-89-3 |
| L-Cysteine | CDAA-270022-200mg | ANPEL | 52-90-4 |
| L-Methionine | CDAA-270011-200mg | ANPEL | 63-68-3 |
| Glycine | ZTO-g0099-25g | TCI | 56-40-6 |
| α-Aminoadipic acid | BD2960-1g | Bidepharm | 1118-90-7 |
| 5-Hydroxy-tryptophan | ZC-28128 | zzstandard | 4350-09-8 |
| Kynurenine | R021003-250mg | Rhawn | 343-65-7 |
| L-Citrulline | C7629-10mg | sigma | 372-75-8 |
| 3-Chloro-L-Tyrosine | R046276-1g | Rhawn | 7423-93-0 |
| L-Alanine | ZTO-A0179-25g | TCI | 56-41-7 |
| L-Arginine | A769505-100mg | TRC | 74-79-3 |
| L-Histidine | ZTO-H0149-25g | TCI | 71-00-1 |
| L-Asparagine Anhydrous | ZCX-00011043-100mg | ChromaDex | 70-47-3 |
| Homoserine | 433471-1g | J&K Scientific | 1927-25-9 |
| N8-Acetylspermidine | ZTR-A188000-50mg | TRC | 34450-15-2 |
| L-Ornithine | CDAA-270021-200mg | ANPEL | 3184-13-2 |
| N'-Formylkynurenine | ZTR-F700490-2.5mg | TRC | 1022-31-7 |
| argininosuccinic acid | 73097-10mg | sigma | 2387-71-5 |
| Creatine Phosphate | R008138-1g | Rhawn | 922-32-7 |
| L-Glutamic acid | G103978-100g | aladdin | 56-86-0 |
| glycylphenylalanine | CFFC-G0136-100mg | TCI | 3321-03-7 |
| L-Lysine | ZTO-L0129-5g | TCI | 56-87-1 |
| S-Sulfo-L-Cysteine | ZC-70242 | zzstandard | 1637-71-4 |
| 5-Hydroxy-Tryptamine | R053990-5mg | Rhawn | 50-67-9 |
| L-Serine | ZTO-S0035-5g | TCI | 56-45-1 |
| γ-Glutamate-Cysteine | ZC-25172 | zzstandard | 636-58-8 |
| L-Tryptophyl-L-glutamic acid | ZC-27204 | zzstandard | 36099-95-3 |
| L-tyrosine methyl ester | CFEQ-4-522746-0001 | CNW | 1080-06-4 |
| L-Theanine | SMB00395-100mg | sigma | 3081-61-6 |
| (S)-β-Aminoisobutyric Acid | ZTR-M276560-10mg | TRC | 4249-19-8 |
| Homo-L-arginine | F-27800005 | BACHEM | 156-86-5 |
| Ethanolamine | ALR-136W | AccuStandard | 141-43-5 |
| 3-Hydroxyhippuric Acid | H943125 | TRC | 1637-75-8 |
| 1,3,7-Trimethyluric Acid | / | zzstandard | 5415-44-1 |
| Anserine | ZR-50007-1g | zzstandard | 584-85-0 |
| Trimethylamine N-Oxide | CFEQ-4-521372-0001 | CNW | 1184-78-7 |
| L-Pipecolic Acid | CFEQ-4-511414-0001 | CNW | 3105-95-1 |
| N,N-Dimethylglycine | CFEQ-4-443595-0001 | CNW | 1118-68-9 |
| N-Acetylneuraminic Acid | CFEQ-4-410649-0250 | CNW | 131-48-6 |
| 3-Aminoisobutanoic Acid | CFEQ-4-410334-0001 | CNW | 144-90-1 |
| γ-Aminobutyric Acid | CFEQ-4-410292-0005 | CNW | 56-12-2 |
| N-Glycyl-L-Leucine | CDDM-G750000-10g | TRC | 869-19-2 |
| L-Homocystine | CDDM-H591360-10g | TRC | 626-72-2 |
| Nicotinuric Acid | CDDM-N433500-250mg | TRC | 583-08-4 |
| Guanidinoethyl Sulfonate | CDDM-G827500-5g | TRC | 543-18-0 |
| 1-Methylhistidine | CDDM-M312050-100mg | TRC | 332-80-9 |
| N-Isovaleroylglycine | CDDM-I917600-2.5g | TRC | 16284-60-9 |
| Nα-Acetyl-L-Arginine | CFAD-A3133-5G | sigma | 155-84-0 |
| Phosphorylethanolamine | P0503-1g | sigma | 1071-23-4 |
| L-Cystathionine | CDDM-C989500-25mg | TRC | 56-88-2 |
| 4-Acetamidobutyric Acid | CFDL-A11039-10g | Alfa Aesa | 3025-96-5 |
| Nα-Acetyl-L-glutamine | CFEQ-4-410794-0005 | cnw | 2490-97-3 |
| L-α-Aspartyl-L-phenylalanine | 421669-100mg | sigma | 13433-09-5 |
| 1,3-Dimethyluric Acid | CFAD-D2889-100mg | sigma | 944-73-0 |
| D-Alanyl-D-Alanine | CFAD-A0912-250mg | sigma | 923-16-0 |
| Glycyl-L-Proline | CFFC-G0137-1G | TCI | 704-15-4 |
| N6-Acetyl-L-Lysine | CFAD-A4021-1G | sigma | 692-04-6 |
| 5-Aminovaleric Acid | T5089 | Topscience | 660-88-8 |
| Methionine Sulfoxide | CFEQ-4-490111-0001 | CNW | 62697-73-8 |
| P-Aminohippuric Acid | CDCU-1025351 | USP | 61-78-9 |
| O-Phospho-L-Serine | CFEQ-4-510783-0001 | CNW | 407-41-0 |
| 3,7-Dimethyluric Acid | CFAF-40409-250mg | sigma | 13087-49-5 |
| L-Carnosine | CDCV-ASB-00003201-100 | ChromaDex | 305-84-0 |
| (5-L-Glutamyl)-L-Amino Acid | CFAE-483834-500mg | sigma | 5875-41-2 |
| N-Acetylaspartate | CFAF-00920-5G | sigma | 997-55-7 |
| 3-Iodo-L-Tyrosine | CFEQ-4-480085-0001 | cnw | 70-78-0 |
| N-Acetyl-L-Tyrosine | CFEQ-4-411419-0005 | cnw | 537-55-3 |
| Sarcosine | CFEQ-4-490342-0005 | cnw | 107-97-1 |
| 2-Aminobutyric acid | CFEQ-4-410836-0001 | cnw | 1492-24-6 |
| Beta-Alanine | CFEQ-4-410190-0005 | cnw | 107-95-9 |
| 6-Aminocaproic Acid | CFEQ-4-412265-0005 | cnw | 60-32-2 |
| D-Homocysteine | 44925-25mg | sigma | 454-29-5 |
| L-Tryptophan | ZES-5569 | ExtraSynthese | 73-22-3 |
| 3-N-Methyl-L-Histidine | ZTR-M312005 | TRC | 368-16-1 |
| L-Isoleucine | ZTO-I0181-25g | TCI | 73-32-5 |
| L-Valine | ZTO-V0014-25g | TCI | 72-18-4 |
| L-Tyrosine | ZTO-T0550-25g | TCI | 60-18-4 |
| Urea | ZTO-U0077-5g | TCI | 57-13-6 |
| 2-Aminoethanesulfonic Acid | ZTO-A0295-25g | TCI | 107-35-7 |
| L-Threonine | ZTO-T0230-100g | TCI | 72-19-5 |
| L-Phenylalanine | ZTO-P0134-25g | TCI | 63-91-2 |
| L-Leucine | ZTO-L0029-25g | TCI | 61-90-5 |
| L-Aspartate | ZTO-A0546-25g | TCI | 56-84-8 |
| Trans-4-Hydroxy-L-Proline | ZTO-H0296-25g | TCI | 51-35-4 |
| L-Proline | ZTO-P0481-25g | TCI | 147-85-3 |
| Creatine | ZDR-C11748500-250mg | Dr.Ehrenstorfer | 57-00-1 |
| L-Homocitrulline | ZR-18879-1g | zzstandard | 1190-49-4 |
| L-Glutamine | CDAA-281494-20mg | ANPEL | 56-85-9 |
| N-Propionylglycine | ZL-170572 | zzstandard | 21709-90-0 |
| Succinic Acid | ZT-20907-100mg | zzstandard | 110-15-6 |
| Glutathione Oxidized | SCRSTANDARD | SCMR-333823 | 121-24-4 |
| 5-Hydroxylysine | ZWK-089-08231 | wako | 1190-94-9 |
| S-(5-Adenosy)-L-homocysteine | CDDM-A291500-50mg | TRC | 979-92-0 |
| Kynurenic Acid | SCMR-300140 | SCRSTANDARD | 492-27-3 |

Table S3. Identification of amino acid composition in boletes using LC-MS.

| Index | Class | RT | LLOQ | ULOQ |
| --- | --- | --- | --- | --- |
| (5-L-Glutamyl)-L-Alanine | Amino Acid metabolomics | 9.56 | 100 | 20000 |
| 1-Methylhistidine | Amino Acid metabolomics | 9.07 | 50 | 20000 |
| 3-Aminoisobutanoic-Acid | Amino Acid metabolomics | 7.27 | 200 | 10000 |
| 3-Chloro-L-Tyrosine | Amino Acid metabolomics | 5.86 | 20 | 20000 |
| 3-Iodo-L-Tyrosine | Amino Acid metabolomics | 5.48 | 10 | 20000 |
| 3-N-Methyl-L-Histidine | Amino Acid metabolomics | 9.86 | 50 | 20000 |
| 5-Hydroxylysine | Amino Acid metabolomics | 10.42 | 20 | 20000 |
| 5-Hydroxy-Tryptamine | Amino Acid metabolomics | 2.9 | 50 | 20000 |
| 5-Hydroxy-tryptophan | Amino Acid metabolomics | 6.97 | 10 | 20000 |
| Ala | Amino Acid metabolomics | 7.99 | 100 | 20000 |
| Arg | Amino Acid metabolomics | 9.96 | 10 | 20000 |
| argininosuccinic-acid | Amino Acid metabolomics | 10.64 | 20 | 20000 |
| Asn | Amino Acid metabolomics | 8.87 | 50 | 20000 |
| Asp | Amino Acid metabolomics | 9.63 | 100 | 20000 |
| Asp-Phe | Amino Acid metabolomics | 8.52 | 10 | 20000 |
| Beta-Alanine | Amino Acid metabolomics | 8.05 | 100 | 20000 |
| D-Alanyl-D-Alanine | Amino Acid metabolomics | 8.59 | 10 | 20000 |
| D-Homocysteine | Amino Acid metabolomics | 6.68 | 500 | 20000 |
| Gln | Amino Acid metabolomics | 8.73 | 50 | 20000 |
| Glu | Amino Acid metabolomics | 9.15 | 10 | 20000 |
| Glutathione-Oxidized | Amino Acid metabolomics | 10.91 | 20 | 20000 |
| Gly | Amino Acid metabolomics | 8.41 | 200 | 20000 |
| Glycyl-L-Proline | Amino Acid metabolomics | 8.86 | 10 | 20000 |
| glycylphenylalanine | Amino Acid metabolomics | 7.3 | 10 | 20000 |
| His | Amino Acid metabolomics | 9.72 | 500 | 20000 |
| Homo-Arg | Amino Acid metabolomics | 9.81 | 10 | 10000 |
| Homoserine | Amino Acid metabolomics | 8.38 | 50 | 20000 |
| Ile | Amino Acid metabolomics | 5.33 | 50 | 20000 |
| L-Carnosine | Amino Acid metabolomics | 10.18 | 50 | 20000 |
| L-Citrulline | Amino Acid metabolomics | 9.08 | 20 | 20000 |
| L-Cystathionine | Amino Acid metabolomics | 10.51 | 10 | 20000 |
| L-Cystine | Amino Acid metabolomics | 10.56 | 10 | 10000 |
| Leu | Amino Acid metabolomics | 5.01 | 100 | 20000 |
| L-Homocitrulline | Amino Acid metabolomics | 8.86 | 10 | 20000 |
| L-Homocystine | Amino Acid metabolomics | 10.15 | 10 | 20000 |
| L-Ornithine | Amino Acid metabolomics | 10.18 | 50 | 20000 |
| L-Pipecolic-Acid | Amino Acid metabolomics | 6.93 | 10 | 5000 |
| L-Theanine | Amino Acid metabolomics | 7.29 | 10 | 20000 |
| L-tyrosine-methyl-ester | Amino Acid metabolomics | 1.52 | 10 | 20000 |
| Lys | Amino Acid metabolomics | 10.13 | 20 | 20000 |
| Met | Amino Acid metabolomics | 6.07 | 1000 | 20000 |
| Methionine-Sulfoxide | Amino Acid metabolomics | 8.7 | 20 | 20000 |
| N,N-Dimethylglycine | Amino Acid metabolomics | 6.46 | 10 | 10000 |
| N6-Acetyl-L-Lysine | Amino Acid metabolomics | 8.15 | 10 | 20000 |
| N-Acetylaspartate | Amino Acid metabolomics | 5.62 | 500 | 10000 |
| N-Acetyl-L-Tyrosine | Amino Acid metabolomics | 2.22 | 10 | 20000 |
| N-Glycyl-L-Leucine | Amino Acid metabolomics | 7.33 | 10 | 10000 |
| Nicotinuric-Acid | Amino Acid metabolomics | 4.58 | 10 | 20000 |
| N-Isovaleroylglycine | Amino Acid metabolomics | 1.61 | 10 | 20000 |
| N-Propionylglycine | Amino Acid metabolomics | 2.8 | 10 | 20000 |
| Nα-Acetyl-L-Arginine | Amino Acid metabolomics | 8.27 | 10 | 20000 |
| Nα-Acetyl-L-glutamine | Amino Acid metabolomics | 7.12 | 20 | 5000 |
| O-Phospho-L-Serine | Amino Acid metabolomics | 10.78 | 2000 | 20000 |
| Phe | Amino Acid metabolomics | 4.9 | 10 | 20000 |
| Pro | Amino Acid metabolomics | 6.72 | 20 | 20000 |
| S-(5-Adenosyl)-L-Homocysteine | Amino Acid metabolomics | 9.06 | 10 | 20000 |
| Ser | Amino Acid metabolomics | 8.78 | 200 | 20000 |
| S-Sulfo-L-Cysteine | Amino Acid metabolomics | 7.82 | 10 | 20000 |
| Succinic-Acid | Amino Acid metabolomics | 1.7 | 100 | 20000 |
| Thr | Amino Acid metabolomics | 8.23 | 100 | 20000 |
| Trans-4-Hydroxy-L-Proline | Amino Acid metabolomics | 7.97 | 100 | 20000 |
| Trimethylamine-N-Oxide | Amino Acid metabolomics | 3.34 | 10 | 20000 |
| Trp | Amino Acid metabolomics | 5.04 | 50 | 20000 |
| TRP-GLU | Amino Acid metabolomics | 8.15 | 10 | 20000 |
| Tyr | Amino Acid metabolomics | 6.99 | 50 | 20000 |
| Urea | Amino Acid metabolomics | 1.54 | 500 | 20000 |
| Val | Amino Acid metabolomics | 6.66 | 100 | 20000 |
| γ-Glutamate-Cysteine | Amino Acid metabolomics | 9.52 | 5000 | 20000 |
